# Effects of the Expressions and Variants of the *CAST* Gene on the Fatty Acid Composition of the Longissimus Thoracis Muscle of Grazing Sonid Sheep

**DOI:** 10.3390/ani13020195

**Published:** 2023-01-04

**Authors:** Xin Guo, Terigele Li, Datong Lu, Takahisa Yamada, Xihe Li, Siqin Bao, Jiasen Liu, Gerelt Borjigin, Ming Cang, Bin Tong

**Affiliations:** 1 The State Key Laboratory of Reproductive Regulation and Breeding of Grassland Livestock, School of Life Sciences, Inner Mongolia University, Hohhot 010021, China; 2 Inner Mongolia Agriculture Animal Husbandry Fishery and Biology Experiment Research Centre, Inner Mongolia Agricultural University, Hohhot 010010, China; 3 Department of Agrobiology, Faculty of Agriculture, Niigata University, Niigata 950-2181, Japan; 4 Institute of Animal Science, Inner Mongolia Academy of Agricultural and Animal Husbandry Sciences, Hohhot 010031, China; 5 College of Food Science and Engineering, Inner Mongolia Agricultural University, Hohhot 010010, China

**Keywords:** association, *CAST* gene, expression, fatty acid, Sonid sheep, variants

## Abstract

**Simple Summary:**

This study aimed to evaluate the relationship between the expression levels of the *CAST* gene and the fatty acid (FA) composition in the longissimus thoracis (LL) muscle and to identify novel variants of *CAST* and perform association analysis with the FA composition in grazing Sonid sheep. The correlation results showed that high expression levels of *CAST* are correlated with better FA compositions and classes in LL. Four c.646G>C (G216R), c.1210C>T (R404C), c.1437G>A (479T), and c.2097C>T (699G) mutations were identified in the *CAST* gene of Sonid sheep. The association studies showed that c.1210C>T is associated with C14:0, C18:0, C18:1n9c, C18:3n3, n3, C12:0, n6, and n6/n3; c.646G>C and c.1437G>A in linkage disequilibrium-Mongolia (*r*^2^ = 0.964) were associated with C14:0, C18:0, SFA, C18:1n9c, n3, C10:0, C18:1n9t, and n6/n3; and c.2097C>T was associated with C18:3n3, n3, C10:0, and n6/n3 in the LL of Sonid sheep. Thus, the correlation results and associated mutations were expected to be genetic selection markers for the FA composition and meat quality of Sonid sheep muscle and provide new insight into sheep meat quality traits influenced by the ovine *CAST* gene.

**Abstract:**

Fatty acid (FA) composition has an important impact on the nutrition and flavor of meat, and on consumer health, and is receiving more attention in the sheep industry. This study aimed to evaluate the relationship between the expression levels of the *CAST* gene and the FA composition in the longissimus thoracis (LL) muscle, to identify novel variants of *CAST*, and to perform association analysis with the FA composition in grazing Sonid lambs. The correlation results showed that high expression levels of *CAST* are correlated with better FA compositions and classes in LL. For association studies, the results showed that c.1210C>T and c.1437G>A in LD-M, and c.2097C>T mutations are associated with some compositions and classes of FA in the LL of grazing Sonid sheep. Two missense c.646G>C (G216R) and c.1210C>T (R404C) mutations were predicted to influence the Calpain_inhib domains of *CAST*. Thus, the correlation results and associated mutations are expected to be genetic selection markers for the FA composition and meat quality of grazing Sonid lamb muscle and provide new insights into sheep meat quality traits influenced by the ovine *CAST* gene.

## 1. Introduction

In China, the annual lamb consumption per person increased from 2.99 kg in 2011 to 3.93 kg in 2021. China has the largest number of sheep in the world, with nearly 200 million before 2022. Among them, the Mongolia sheep population is the most widely distributed and the most numerous. Mongolia sheep, an old and primitive sheep breed, is mainly distributed in the grassland, desert, and agricultural areas in northern China, Mongolia, and Central Asia and is the common ancestor of Chinese short fat-tailed sheep breeds, such as the Sonid, Ujimqin, Hulunbuir, Tan, Bayanbulak, Small-tailed Han, Duolang, and Hu sheep (China National Commission of Animal Genetic Resources) [1]. Recently, the meat of Mongolia sheep populations was recognized as a natural green food and has become increasingly popular in China.

The fatty acid (FA) composition of lamb meat is an important trait for the determination of flavor and nutrition, and this meat can provide essential FA for human health [2]. The types and levels of FA are influenced by polygenic and environmental factors [3,4]. A better knowledge of the molecular architecture of meat FA composition is important as it may generate new opportunities for more effective marker-assisted breeding, leading to economic benefits for the sheep industry [5]. Nevertheless, compared to beef and pork, the studies of genetic effects, including the mutation and expression of candidate genes, on the FA composition of lamb are few.

Calpastatin (CAST) is involved in the calpain–calpastatin system, which influences many important processes, including muscle development and growth [6], and is also known to regulate the degradation of myofibrillar proteins both in living and in post-mortem muscle tissue [7,8]. To date, many polymorphisms of the *CAST* gene have been identified and associated with meat quality traits, such as tenderness, color, and intramuscular fat (IMF) content, as well as carcass traits in cattle and pigs [9,10,11,12,13,14]. However, research focusing on the relationship between the *CAST* gene and meat quality, especially FA composition, in sheep is limited. A report showed that a polymorphic variant by PCR-SSCP in intron 12 of the ovine *CAST* gene is associated with IMF content in two Polish synthetic lines of sheep [15]. Therefore, the *CAST* gene could be considered a potential candidate gene for meat quality traits in sheep.

Thus, the aims of this study reported herein were to (1) evaluate the effects of the expression levels of *CAST* on the FA composition in the longissimus thoracis muscle of sheep, (2) identify novel variants of *CAST* and perform association analysis with the FA composition in Sonid sheep, and (3) predict the effect of the novel variants on the feature and structure of the mRNA and protein of the ovine *CAST* gene. Our study may facilitate effective marker-assisted selection to promote the meat quality in Mongolia sheep populations and provide new insights into the effect of the ovine *CAST* gene on sheep meat quality traits.

## 2. Materials and Methods

### 2.1. Animals and Samples

The study was carried out on 378 castrated ram lambs (approximately 6 months old) of Sonid sheep, born in 2020. The lambs were raised until weaning in an outdoor extensive grazing system, feeding on natural pastures as usual in the Sonid grassland of Inner Mongolia. After weaning, the castrated male lambs were grazed under the same conditions as usual in the Sonid grassland until 6 months old. All the experimental animals were healthy. The 378 lambs were the progeny of different sires and dams, and any relationship between the parents was not recorded. These lambs were the progeny of more than 20 unrelated sires.

Lambs were slaughtered in accordance with the Chinese industry standard (NY/T 1564-2021). After being slaughtered, the longissimus thoracis (LL) muscle tissues (at least 500 g between the 12 and 13th ribs) of 378 animals (left half of the carcass) were removed on ice, taken to the laboratory, frozen, and stored at −35 °C until analysis for FA composition. Of the 378 lambs, the LL samples of 90 animals were randomly selected and frozen in liquid nitrogen immediately after slaughter and stored at −80 °C until used for RNA extraction. Three lambs were randomly selected to sample the LL, semitendinosus, heart, subcutaneous fat, liver, lung, spleen, kidney, large intestine, small intestine, and stomach, which were taken and stored in liquid nitrogen for tissue expression profile analysis of the *CAST* gene.

### 2.2. Determination of Fatty Acid Composition

The fatty acid profile was determined after extracting the total lipids and performing esterification and methylation processes using the method in accordance with Folch et al. [16]. After the extracted lipid was converted to fatty acid methyl esters (FAMEs), the FA profiles were analyzed using a gas chromatography–flame ionization detector (Varian 450-GC, Bruker Daltonics Inc., Fremont, CA, USA). Injector and detector temperatures were held at 260 °C. The oven temperature was initially 120 °C for 5 min and was increased to 230 °C at 3 °C/min and held at that temperature for 3 min, and then increased to 240 °C at a rate of 1.5 °C/min and held for 5 min. The samples containing FAMEs in hexane (1.0 μL) were injected through the split injection port (10:1) onto an RT-2560 capillary column (100 m length, 0.25 mm internal diameter, 0.20 μm film thickness; RESTEK, Bellefonte, PA, USA). Individual FAMEs were identified by comparing their retention times with those of authenticated standards, and the results were expressed as a percentage of the total FAMEs. In addition, individual FAs were used to calculate the sums of saturated fatty acids (SFA), monounsaturated fatty acids (MUFA), and polyunsaturated fatty acids (PUFA).

### 2.3. RNA Extraction and Real-Time Quantitative Amplification

Total RNA from the LL of 90 Sonid sheep was extracted using RNAiso Plus (Takara Bio Inc., Dalian, China), and cDNA synthesis was performed using a PrimeScript^TM^ RT Reagent Kit with gDNA Eraser (Takara Bio Inc., Dalian, China) according to the manufacturer’s protocol. Real-time polymerase chain reaction (PCR) analyses were performed on a BIO-RAD Real-time PCR system in a 20 μL reaction volume, including 10 μL of 2 × SYBR^®^ Premix Ex Taq^TM^II (Takara Bio Inc., Dalian, China), 0.8 μL of forward and reverse primers (10 μL), 0.4 μL of ROX Reference Dye, 2.0 μL of template cDNA, and 6.0 μL of nuclease-free water. The real-time PCR conditions were as follows: 95 °C for 30 s, 40 cycles of 95 °C for 5 s, and 60 °C for 34 s. We tested the gene suitability of the glyceraldehyde-3-phosphate dehydrogenase (*GAPDH*) gene as the internal housekeeping gene in this experiment. The gene expression stability value was less than 0.05, which met the stability required to be a housekeeping gene [17]. The expression of each gene was normalized against *GAPDH* (Table 1), and relative expression levels were determined using the 2^−∆∆Ct^ method [18]. 

### 2.4. Resequencing and Variant Detection in CAST

Genomic DNA was isolated from the LL using the Wizard^®^ Genomic DNA Purification Kit (Promega Corp., Madison, WI, USA). The quality and quantity of the extracted DNA were tested with a NanoDrop 1000 spectrophotometer (Thermo Fisher Scientific, Waltham, MA, USA). The quality of the genomic DNA preparations was also verified using agarose gel electrophoresis. Genomic DNA with absorbance at OD260 nm/OD280 nm of ≥1.80 and no RNA or protein in the agarose gel was used as a template. The DNA of 10 Sonid sheep was randomly selected and sent to Beijing Novogene Company for 10 × genome resequencing.

### 2.5. Polymorphism Genotyping Using iPLEX MassARRAY

Four target variants were genotyped with the MassARRAY^®^ SNP genotyping system (Agena Bioscience, San Diego, CA, USA) in the 378 Sonid sheep. PCR and extension primers were designed from sequences containing each target mutation and ~100 upstream and downstream bases with Assay Design Suite (http://agenabio.com/assay-design-suite-20-software) using the default settings (Table 2). The genotype of each SNP was analyzed using the Sequenom MassARRAY iPLEX platform (Sequenom, San Diego, CA, USA) [19]. The resulting data were analyzed using MassARRAY Typer 4.0 Analyzer software (Agena Bioscience, San Diego, CA, USA).

### 2.6. Bioinformatics Analysis

Alignment of the wild-type sequence in various species of mutations in the coding region of the *CAST* gene was performed with Clustal Omega using UniProt (http://www.uniprot.org, accessed on 13 June 2022) online tools. Using ProtParam, we predicted the fundamental properties of CAST proteins (http://www.expasy.org/tools/protparam.html, accessed on 16 June 2022). The transmembrane domains were speculated using TMHMM (https://services.healthtech.dtu.dk/service.php?TMHMM-2.0, accessed on 16 June 2022). SMART (Simple Molecular Architecture Research Tool; http://smart.embl-heidelberg.de/, accessed on 16 June 2022) was used to forecast the conserved domain of *CAST*. Using NetNGlyc (https://services.healthtech.dtu.dk/service.php?NetNGlyc-1.0, accessed on 16 June 2022) and NetPhos (https://services.healthtech.dtu.dk/service.php?NetPhos-3.1, accessed on 16 June 2022), we predicted phosphorylation and N-glycosylation sites. ProtScale (http://web.expasy.org/protscale/, accessed on 16 June 2022) was used to determine the hydrophilicity and average flexibility index of ovine CAST amino acid sequences. The secondary structure of the sheep’s *CAST* gene was predicted using RNAfold (http://rna.tbi.univie.ac.at/cgi-bin/RNAWebSuite/RNAfold.cgi, accessed on 18 June 2022). SOPMA was used to predict the secondary structure of the ovine CAST protein (https://npsa-prabi.ibcp.fr/cgi-bin/npsa_automat.pl?page=npsa_sopma.html, accessed on 18 June 2022). The tertiary structure of ovine CAST was predicted using SWISS-MODEL (https://www.swissmodel.expasy.org/, accessed on 19 June 2022) and AlphaFold (https://alphafold.ebi.ac.uk/, accessed on 19 June 2022) [20,21]. Multiple alignments and molecular phylogenetic tree construction were performed using NCBI Blast (https://blast.ncbi.nlm.nih.gov/Blast.cgi, accessed on 13 June 2022) and MEGA-X.

### 2.7. Statistical Analysis

Genotypic and allelic frequencies and Hardy–Weinberg equilibrium were calculated for the Sonid sheep in this study. Population genetic indices, observed heterozygosity (H_o_), expected heterozygosity (H_e_), effective allele numbers (n_e_), and the polymorphism information content (PIC) were calculated using Nei’s methods [22]. The allelic frequency of each variant was compared using a χ^2^ test. The LD, including D’ and *r*^2^, was assessed using HaploView 4.2 software [23]. Haplotypes were obtained using SHEsis [24]. The associations between FA composition/class and different genotypes of the four novel variants and three haplotypes in the Sonid sheep were analyzed using SPSS 24.0 (SPSS, Inc., Chicago, IL, USA). The statistical linear model was as follows: Y_i_ = μ + G_i_ + e_i_, where Y_i_ is the observed value of fatty acid composition traits, μ is the mean of each measurement, G_i_ means the fixed effect of the genotypes, and e_i_ means standard error. When the number of sheep with a given genotype was less than 10, their associations and effects could not be reliably estimated. Therefore, animals with this genotype were excluded from the analysis. The Bonferroni correction was used to adjust *p* values [25]. Correlation analyses between any two traits of FA compositions and FA classes, and between expression levels of *CAST* and each of the FA compositions and FA classes, were calculated using SPSS 24.0, and results are presented as the Pearson correlation coefficient (SPSS, Inc., Chicago, IL, USA).

## 3. Results

### 3.1. Fatty Acid Profiles of the Longissimus Thoracis Muscle

**Figure 1:**
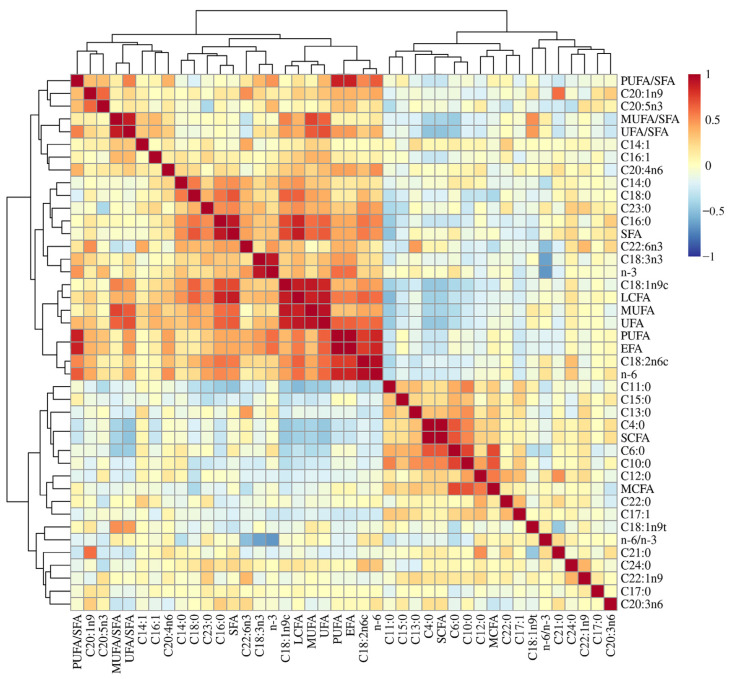
Correlogram of the correlation analyses between any two traits of FA compositions and classes. The correlation matrix was reordered according to the correlation coefficient, aiming to make the clusters of correlated variables more evident. Positive correlations are displayed in red and negative correlations in blue color. The color intensity and size of circles are proportional to the size of the correlation coefficients.

The analyzed FA compositions and FA classes in the LL of the 378 Sonid sheep are listed in Table S1, including 15 kinds of SFA, 7 kinds of MUFA, and 6 kinds of PUFA. The correlation analyses between any two traits of FA compositions and FA classes are graphically represented in Figure 1, and the correlation coefficients and relative *p* values are reported in Table S2. For SFA, the LL content of capric acid (C10:0) was strongly correlated with caproic acid (C6:0), undecanoic acid (C11:0), tridecanoic acid (C13:0), butyric acid (C4:0), and lauric acid (C12:0), with *r* values ranging from 0.748 to 0.454 and *p* < 0.001 (Figure 1, Table S2). The content of C4:0 was strongly correlated with C6:0, C11:0 was strongly correlated with pentadecanoic acid (C15:0), and tricosanoic acid (C23:0) was strongly correlated with tetracosanoic acid (C24:0), with *r* values ranging from 0.680 to 0.292 and *p* < 0.001 (Figure 1, Table S2). The content of palmitic acid (C16:0) was positively correlated with stearic acid (C18:0), C23:0, and myristic acid (C14:0), with *r* values ranging from 0.590 to 0.485 and *p* < 0.001 (Figure 1, Table S2). The content of C18:0 was positively correlated with C14:0 and C23:0, with *r* values ranging from 0.567 to 0.387 and *p* < 0.001 (Figure 1, Table S2). In contrast, the correlation analyses of unsaturated fatty acid (UFA) revealed that linoleic acid (C18:2n6c) showed significant correlations with oleic acid (C18:1n9c), eicosenoic acid (C20:1n9), and arachidonic acid (C20:4n6), and the content of C18:1n9c was strongly correlated with α-linolenic acid (C18:3n3), with *r* values ranging from 0.484 to 0.266 and *p* < 0.001 (Figure 1, Table S2). In addition, C18:1n9c showed strong positive correlations with C16:0, C18:0, C14:0, and C23:0, with *r* values ranging from 0.723 to 0.451 and *p* < 0.001 (Figure 1, Table S2). The content of C18:2n6c was positively correlated with C16:0, C23:0, C18:0, and C24:0, with *r* values ranging from 0.579 to 0.362 and *p* < 0.001 (Figure 1, Table S2). The content of C18:3n3 was positively correlated with C23:0 and C16:0, with *r* values ranging from 0.369 to 0.299 and *p* < 0.001 (Figure 1, Table S2).

Meanwhile, the content of C18:1n9c showed strong negative correlations with C12:0, C13:0, C15:0, C10:0, C6:0, C11:0, and C4:0, with *r* values ranging from −0.238 to −0.467 and *p* < 0.01 (Figure 1, Table S2). The content of C11:0 showed strong negative correlations with C18:3n3 and eicosapentaenoic acid (C20:5n3), with *r* values ranging from −0.261 to −0.357 and *p* < 0.01 (Figure 1, Table S2). The content of C18:2n6c showed strong negative correlations with C15:0, C13:0, and C11:0, with *r* values ranging from −0.188 to −0.383 and *p* < 0.01 (Figure 1, Table S2). The content of C16:0 showed strong negative correlations with ginkgolic acid (C17:1) and elaidic acid (C18:1n9t), with *r* values ranging from −0.171 to −0.228 and *p* < 0.01 (Figure 1, Table S2). 

### 3.2. CAST Gene Expression Profiles in Sheep

**Figure 2:**
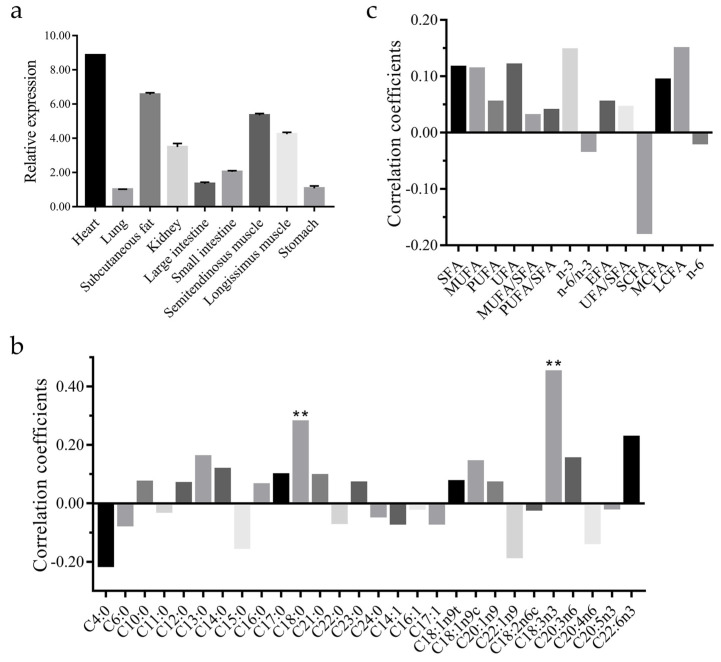
*CAST* gene expression profiles in sheep. (**a**) Relative expression levels of the *CAST* gene in nine different tissues of Sonid sheep (*n* = 3). (**b**) Correlations between fatty acid composition and expression levels of *CAST* in the longissimus thoracis muscle of 90 Sonid sheep. (**c**) Correlations between fatty acid classes and expression levels of *CAST* in the longissimus thoracis muscle of 90 Sonid sheep. ** *p* < 0.01.

*CAST* gene expression profiles in various tissues of Sonid sheep are shown in Figure 2a. *CAST* gene expressions were significant higher in heart tissue, subcutaneous fat, the LL, and semitendinosus muscle than in other tissues (Figure 2a; statistical difference of the *CAST* gene expression between different tissues are shown in Table S3). *CAST* gene expressions were significantly positively correlated with C18:0 and C18:3n3 (*p* < 0.01; Figure 2b,c). 

### 3.3. Variant Discovery in the CAST of Sonid Sheep

**Figure 3:**
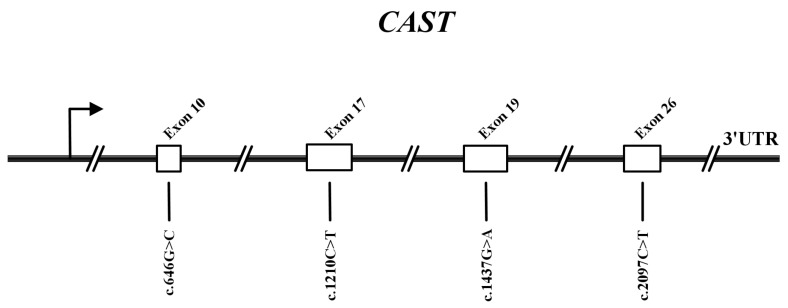
The physical locations of the four novel variants of the ovine *CAST* gene identified in this study are shown. The variant sites were according to chromosome 5 in Oar_rambouillet_v2.0 (GenBank accession: NM_001009788.1).

Sequence analysis revealed four novel variants in the *CAST* gene of Sonid sheep. Among them, the c.646G>C, c.1210C>T, c.1437G>A, and c.2097C>T variants are in exons 10, 17, 19, and 26 of *CAST*, respectively (Figure 3).

**Figure 4:**
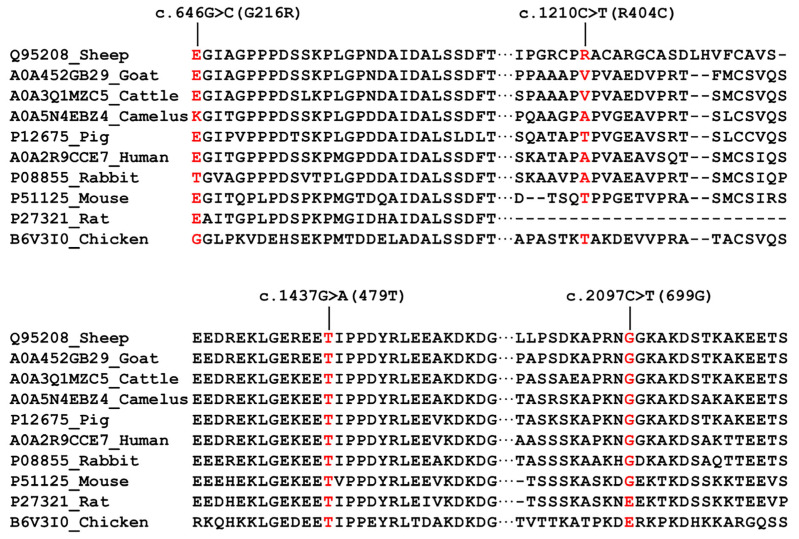
Alignment of the wild-type sequence in various species in the CAST amino acid. CAST multispecies alignment in the region of missense mutations. Using the Uniprot database, CAST amino acid sequences were obtained for each species.

The c.646G>C and c.1210C>T mutations in exons 10 and 17 of *CAST* caused missense mutation at the 216 (glycine to arginine) and 404 (arginine to cysteine) positions in the amino acid sequence of the CAST protein, respectively. The c.1437G>A and c.2097C>T mutations in exons 19 and 26 of *CAST* caused silent mutation at the 479 (threonine) and 699 (glycine) positions in the amino acid sequence of the CAST protein, respectively (Figure 4). 

For each variant, the frequencies of the two alleles and three genotypes in the MG breed are listed in Table S4, as are the genetic indices (H_o_, H_e_, n_e_, PIC, and Hardy–Weinberg equilibrium). The results of the population diversity test showed that four polymorphic loci of the *CAST* gene were low polymorphic or moderate polymorphic in Sonid sheep. No significant departures at the 5% level were detected by any test for each variant in Sonid sheep. The values of the PIC of the three variants (c.646G>C, c.1437G>A, and c.2097C>T) presented with related low polymorphism in Sonid sheep. The values of the PIC of the c.1210C>T variant presented with related moderate polymorphism in the Sonid sheep population (Table S4).

### 3.4. Linkage Disequilibrium Analysis of Novel Variants of CAST

**Figure 5:**
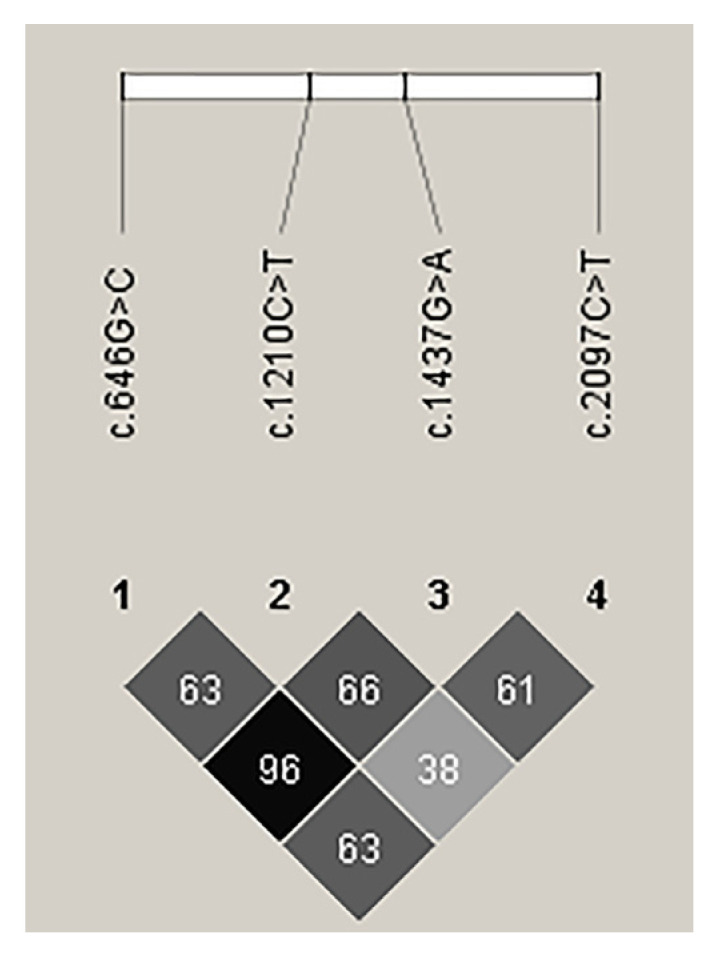
Linkage disequilibrium estimated among *CAST* variations in Sonid sheep. 1: c.646G>C, 2: c.1210C>T, 3: c.1437G>A, 4: c.2097C>T.

To identify the linkage relationships among the four variants, D’ and *r*^2^ were estimated for the experimental Sonid sheep. The c.646G>C and c.1437C>T variants were in nearly complete LD in the experimental Sonid sheep by *r*^2^ = 0.964 (Figure 5). Thus, these LD groups were analyzed together and marked as a single locus, designated LD-M. D’ and *r*^2^ for the experimental Sonid sheep are shown in Table S5.

### 3.5. Associations between Variants in CAST and Fatty Acid

The significant effects of the four novel variants of the *CAST* gene on FA compositions and FA classes in the LL of Sonid sheep are shown in Table 3, and other results are presented in Table S6. For c.1210C>T, the contents of C14:0 (*p* < 0.01), C18:0 (*p* < 0.05), C18:1n9c (*p* < 0.05), and C18:3n3 (*p* < 0.05) of the CC genotype individuals were significantly higher than those of the CT genotype individuals; the contents of n3-PUFA of the CC genotype individuals were significantly higher than those of the CT and TT genotype individuals (*p* < 0.01); the content of C12:0 of the CC genotype individuals was significantly lower than that of CT genotype individuals (*p* < 0.05); the content of n6-PUFA of the CC genotype individuals was significantly lower than that of TT genotype individuals (*p* < 0.01); and the ratio of n6/n3-PUFA of the CC genotype individuals was significantly lower than that of the CT and TT genotype individuals (*p* < 0.01; Table 3). For c.1437G>A in LD-M, compared to the GA genotype, the GG genotype individuals had significantly higher contents of C14:0 (*p* < 0.01), C18:0 (*p* < 0.01), SFA (*p* < 0.05), C18:1n9c (*p* < 0.05), and n3-PUFA (*p* < 0.01), and the GG genotype individuals had significantly lower C10:0 (*p* < 0.05) and C18:1n9t (*p* < 0.05) contents and the ratio of n6/n3-PUFA (*p* < 0.01; Table 3). For c.2097C>T, sheep with the CC genotype had significantly higher C18:3n3 and n3-PUFA levels when compared to sheep with the CT genotype (*p* < 0.05), and the content of C10:0 and the ratio of n6/n3-PUFA of the CC genotype individuals were significantly lower than those of the CT genotype individuals (*p* < 0.05 and *p* < 0.01, respectively) in the LL of Sonid sheep (Table 3).

### 3.6. Associations between Haplotypes of CAST and Fatty Acid

Using the online tool SHEsis, different haplotypes were constructed in the experimental population of Sonid sheep in order to analyze the associations between FA composition and haplotypes. A haplotype with a frequency of >3% was considered a distinguishable haplotype, while haplotypes with a relative frequency of <3% were pooled into a single group. Thus, haplotype 1 (GCGC, H1) had the highest frequency (0.798), followed by haplotype 2 (CTAT, H2, 0.101) and haplotype 3 (GTGC, H3, 0.053); see Table 4. 

Table 4 shows the significant effects of the haplotype of the *CAST* gene on FA composition and FA classes in the LL of Sonid sheep, and other results are presented in Table S7. By haplotype-based association analyses, we found that the content of C12:0 of the H1H1 haplotype individuals was significantly lower than that of the H1H3 haplotype individuals (*p* < 0.05), and the content of heneicosylic acid (C21:0) of the H1H3 haplotype individuals were significantly higher than that of H1H1 (*p* < 0.01) and H1H2 (*p* < 0.05) haplotype individuals (Table 5). Compared to the H1H2 haplotype, the H1H1 haplotype individuals had significantly higher contents of C18:3n3 (*p* < 0.05) and n3-PUFA (*p* < 0.01; Table 5). The ratio of n6/n3-PUFA of the H1H1 haplotype individuals was significantly lower than that of H1H2 (*p* < 0.05) and H1H3 (*p* < 0.05) haplotype individuals (Table 5).

### 3.7. Bioinformatics analysis of ovine CAST

#### 3.7.1. Feature and Structure Prediction of the Ovine CAST Protein

**Figure 6:**
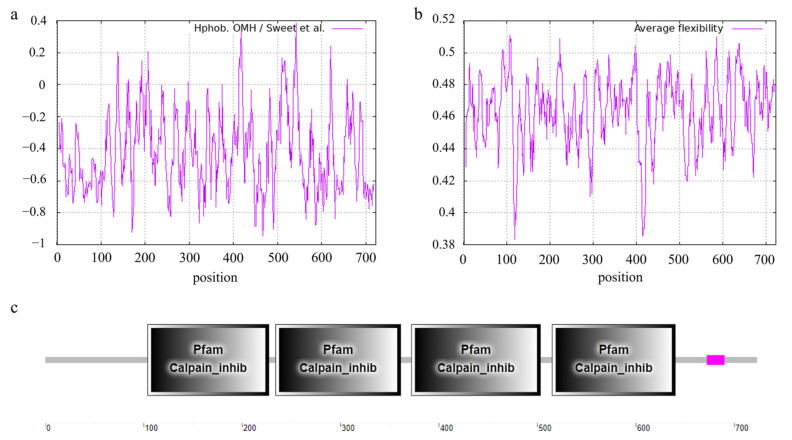
Feature and structure prediction of the ovine CAST protein. (**a**) CAST protein hydrophilicity was analyzed using ProtScale. (**b**) CAST protein average flexibility index was analyzed using ProtScale. (**c**) CAST protein conservative domains were predicted with SMART.

Hydrophobicity analysis of the ovine CAST protein indicated that the maximum hydrophobicity value was 2.189 in the position of 542 aa and the minimum was −3.544 in the position of 25 aa (Figure 6a). The maximum and minimum average flexibility index values of ovine *CAST* were 0.511 for the 107 aa position and 0.383 for the 118 aa position, respectively (Figure 6b). SMART was used to predict the conservative domain. There were four Calpain_inhib domains at 104–227, 234–361, 372–503, and 515–640 aa, and one low-complexity region at 672–690 aa (Figure 6c).

#### 3.7.2. Amino Acid Sequence Analysis of Ovine CAST

ProtParam was used to predict the physicochemical properties of amino acid sequences. The molecular weight and isoelectric point of ovine CAST were 78,998.90 Da and 6.08, respectively. The amino acid composition of the CAST protein showed that the highest proportion of the CAST protein was 13.4% for lysine and the lowest was 0.1% for tryptophan.

**Figure 7:**
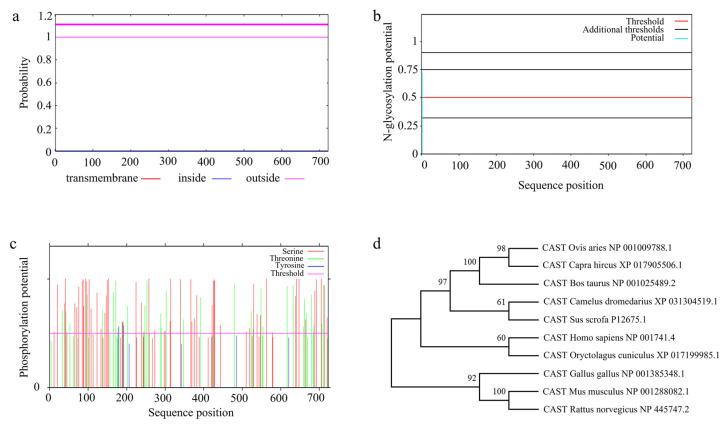
Amino acid sequence analysis of ovine CAST and its multiple sequence alignments. (**a**) CAST protein transmembrane helices were predicted. (**b**) CAST protein N-glycosylation sites were predicted. (**c**) CAST protein phosphorylation sites were predicted. (**d**) A phylogenetic tree was constructed based on CAST homology amino acid sequences.

TMHMM was used to predict subcellular localization based on the protein function and the physical and chemical environment in vivo. There was no transmembrane helix position on the CAST protein (Figure 7a). We predicted 1 N-glycosylation site (Asn) and 95 phosphorylation sites (composed of 59 Ser, 34 Thr, and 2 Tyr) in *CAST* (Figure 7b,c).

#### 3.7.3. Multiple Sequence Alignment and Phylogenetic

To construct phylogenetic trees for the gene identified as homologous to ovine CAST, the amino acid sequence of CAST in some models and domesticated animals was used. After that, a molecular phylogenetic tree was constructed using the maximum-parsimony method, and 1000 Bootstrap replications were carried out using MEGA software (Version X). 

#### 3.7.4. Effect of Variants on the mRNA Secondary Structure of the Ovine CAST Gene

**Figure 8:**
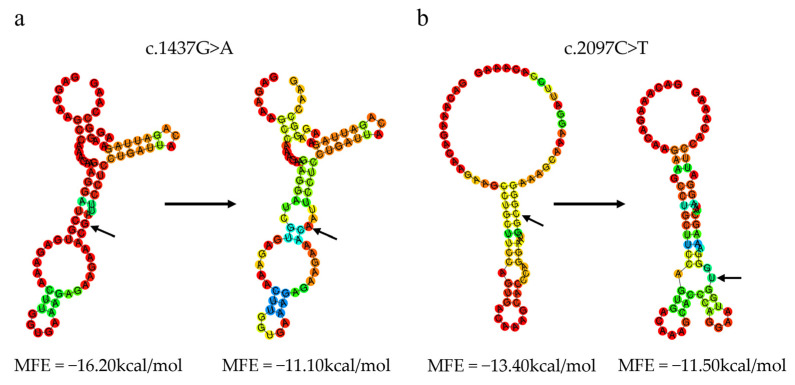
The minimum free energy (MFE) of secondary structure in exons 19 and 26 of the *CAST* gene. MFE prediction in terms of the secondary structure and free energy. (**a**) The secondary structure for the wild-type mRNA sequence of exon 19 with the G allele at the c.1437G>A site and the secondary structure for the mRNA sequence of exon 19 with the A allele at the c.1437G>A site. (**b**) The secondary structure for the wild-type mRNA sequence of exon 26 with the C allele at the c.2097C>T site and the secondary structure for the mRNA sequence of exon 26 with the T allele at the c.2097C>T site. The structure above is colored according to base-pairing probabilities.

Using minimum free energy (MFE)-based RNAfold platform analysis, the results can show a difference in the secondary structure of a point mutation [26]. The MFE of exonic mRNA sequences with wild types at the c.1437G>A in exon 19 and c.2097C>T in exon 26 were −16.20 kcal/mol and −13.40 kcal/mol, respectively. The MFE of the c.1437G>A and c.2097C>T mutations reduced to −11.10 kcal/mol and −11.50 kcal/mol, respectively. As a result of these mutations, the mRNA secondary structure of *CAST* also changed (Figure 8).

#### 3.7.5. Effect of Variants on the Secondary and Tertiary Structure of the Ovine CAST Protein

**Figure 9:**
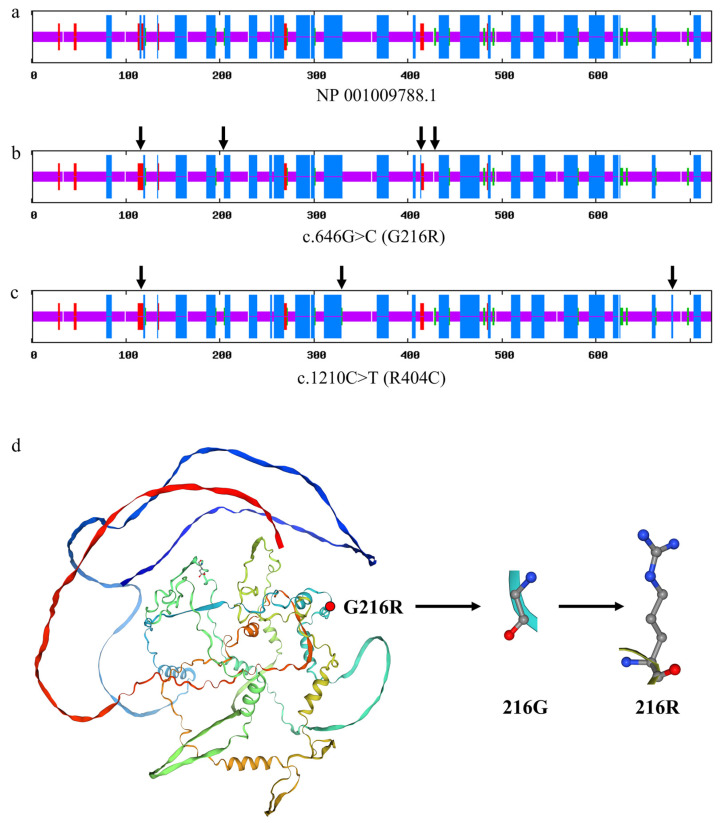
Secondary and tertiary structural changes of the ovine CAST protein. (**a**) Wild-type ovine CAST coding protein secondary structure. (**b**) The protein’s secondary structure changes when glycine changes to arginine at the c.646G>C (G216R) locus. (**c**) The protein’s secondary structure changes when arginine changes to cysteine at the c.1210C>T (R404C) locus. The long blue vertical line represents the alpha helix. The red line represents the extended strand. The short green line represents the beta turn. The purple line represents the random coil. The black arrow indicates where the secondary structure changes. (**d**) The prediction of ovine *CAST* tertiary structures. It was predicted that the amino acid change at the G216R (c.646G>C) site would cause visible changes in the CAST structure.

SOPMA software was used to analyze the secondary structures of the coding proteins of ovine CAST, and the results showed that the percentages of the alpha helix, beta turn, random coil, and extended strand of CAST (CAST Protein ID: NP_001009788.1) were 31.12%, 2.90%, 63.49%, and 2.49%, respectively (Figure 9a). The black arrows indicate four changes in the secondary structure of the protein when the sequence of amino acids changed from glycine to arginine at the c.646G>C (G216R) locus (Figure 9b), and the percentages of the alpha helix, beta turn, random coil, and extended strand of CAST changed to 31.26%, 2.49%, 63.62%, and 2.63%, respectively. The black arrows indicate three changes in the secondary structure of the protein when the amino acid sequence changed from arginine to cysteine of at the c.1210C>T (R404C) locus. (Figure 9c), and the percentages of the alpha helix, beta turn, random coil, and extended strand of CAST changed to 31.40%, 3.04%, 62.79%, and 2.77%, respectively. 

AlphaFold and SWISS-MODEL were used to predict the tertiary structure of the ovine CAST protein (UniProtKB: Q95208). The calcium-dependent complex between calpain-2 and calpastatin (sequence identity 67.06%) was used as a template for modeling (PDB ID: 3DF0.1.C). It was predicted that the amino acid change at the G216R (c.646G>C) site would cause visible changes in the CAST structure (Figure 9d).

## 4. Discussion

The *CAST* gene has been considered a major functional gene related to carcass traits and meat quality in cattle and pig [9,10,11,12,13,14]. However, studies on associations of the expression levels and genetic variants of *CAST* with FA in sheep are rare. In this study, the results of *CAST* expression analyses showed that the expression levels of *CAST* are significantly positive correlated with C18:0 and C18:3n and show a trend of positive correlation with n3, medium-chain fatty acids (MCFA), and long-chain fatty acids (LCFA). In contrast, the expression levels of *CAST* showed a trend of a negative correlation with n6, short-chain fatty acids (SCFA), and n6/n3. Therefore, the high expression levels of *CAST* are more desirable for a better FA composition. Sheep meat generally contains higher levels of SFA, which are widely correlated with health problems, such as heart disease, stroke, and obesity [27], so consumers favor leaner meats containing less SFA and high levels of PUFA [28,29]. PUFA, mainly long-chain omega-3 FAs, are considered beneficial for human health as they reduce serum low-density lipoprotein-cholesterol and total cholesterol and modulate immune functions [30]. Additionally, the desirable sensorial characteristic of meat is associated with PUFA and MUFA [31]. Importantly, sheep meat is rich in omega-3 FA, which is beneficial for human health and immunity [32]. Meat production with a higher PUFA and lower SFA content is, therefore, important to improve human health without requiring substantial changes in customers’ habits of meat consumption [33]. Thus, based on the results of expression analyses, the *CAST* gene could be considered an important candidate gene for a better FA composition and class of meat quality in the sheep industry.

The missense c.646G>C (G216R) and c.1210C>T (R404C) mutations, as well as the silent c.1437G>A (479T) and c.2097C>T (699G) mutations, are the first variants in the *CAST* gene to have a confirmed association with FA in sheep. The amino acid sequence is not altered by synonymous mutations, but they can affect mRNA expression, splicing, stability [34,35,36], and secondary structure [37,38], as well as protein translation, folding [39], and function [40]. In this study, the MFE of the silent c.1437G>A (479T) and c.2097C>T (699G) mutations changed from −16.20 kcal/mol to −11.10 kcal/mol and −13.40 kcal/mol to −11.50 kcal/mol, respectively. As MFE decreases, mRNA’s secondary structure could become more stable [37,38]. Additionally, the c.1437G>A (479T) and c.2097C>T (699G) mutations are predicted to change the mRNA secondary structure of *CAST*. The secondary structure and stability of mRNA can be altered by even single base-pair exchanges, according to some studies [41,42]. According to the results of association studies and bioinformatics analysis, the synonymous c.1437G>A (479T) and c.2097C>T (699G) mutations might be potentially functional mutations in *CAST* for the meat quality of sheep. They could affect the FA synthesis in sheep by altering the mRNA stability and secondary structure of *CAST*. However, further experiments are required to test this hypothesis.

Among the two missense mutations (c.646G>C (G216R) and c.1210C>T (R404C)) identified in this study, c.646G>C (G216R) was modeled in the tertiary structure of the CAST protein, and it was predicted that a visible structure change would occur. Both c.646G>C (G216R) and c.1210C>T (R404C) are located within the Calpain_inhib domain (104–227 aa and 372–503 aa, respectively), which is a conserved domain predicted by SMART. CAST plays an important role in the calpain–calpastatin system, which is an endogenous calcium-dependent proteinase system that mediates the proteolysis of key myofibrillar proteins during the postmortem storage of carcasses and tenderization [7,8,43]. Calpain can be inhibited by CAST, preventing calpain proteolytic activation, membrane binding, and catalytic activity [44]. This may be confirmed by the predominant isoform of CAST, which contains four inhibitory domains (Figure 6c), each of which may inhibit calpain activity, with one molecule of calpastatin able to inhibit four calpain molecules [45]. Therefore, we hypothesize that the missense mutations c.646G>C (G216R) and c.1210C>T (R404C) might change the structure of two Calpain_inhib domains to influence the interaction with calpain, which results in the dynamic change in FA synthesis and FA metabolism in sheep. Of course, this hypothesis still requires further experiments.

Based on the results of the expression and association analyses in this study, the *CAST* gene could be considered an important candidate gene for better FA compositions and meat quality of sheep. FA composition is influenced by diet, genetics, breed, sex, and environmental factors [46,47]. Nevertheless, FA composition is the well-defined compounds describing phenotypic traits, which are possible to improve through genetic selection. FA compositions show moderate-to-high heritability ranging from 0.15 to 0.63 [48,49]. Identification of genetic factors controlling FA composition could be implemented in breeding programs to select animals that produce higher PUFA and lower SFA in meat. Hence, the c.646G>C (G216R), c.1210C>T (R404C), c.1437G>A (479T), and c.2097C>T (699G) mutations of *CAST* identified in this study could be considered useful molecular markers for the purpose of optimizing the composition and class of FA in sheep breeding and industry.

## 5. Conclusions

In summary, this study revealed that the high expression levels of the *CAST* gene are positively correlated with the better FA composition and FA class of grazing Sonid lambs and indicated significant associations between the c.646G>C (G216R), c.1210C>T (R404C), c.1437G>A (479T), and c.2097C>T (699G) mutations of the *CAST* gene and the FA composition and class of grazing Sonid lambs. These association results give breeders more and higher possibilities to select sheep by different favorable alleles of these markers in the breeding of Sonid sheep. Therefore, these findings suggest that the favorable allele in each mutation of the *CAST* gene could be a potentially useful genetic marker for breeding programs aimed at improving the FA composition and class of meat quality of Sonid sheep.

## Figures and Tables

**Table 1:** The primers of *GAPDH* and *CAST* genes for real-time PCR.

Gene	Primer Sequences (5′-3′)	Product Length (bp)	Annealing Temperature (°C)	Accession Number
*GAPDH*	F: AATACTGAGATGTCCTTC	140	53.8	NM_001190390.1
	R: TTTATGGTGGTTGATTTC			
*CAST*	F: ATCCAGAAGACGGAAAGCCT	144	61.3	NM_001009788.1
	R: GCAGTGGTTTTCCGTCTTTATCCTT			

Note: F: forward primer; R: reverse primer.

**Table 2:** Primer sequence information of the variant locus of the *CAST* gene.

Polymorphism	Primers (5′-3′)	Tm (°C)
c.646G>C (G216R)	F: ACGTTGGATGCTTGCCTTCTCAGCATCATC	52.2
	R: ACGTTGGATGTAAACTCACCGAGGAGTCTG	
	E: GAGGCCCTGCGATCC	
c.1210C>T (R404C)	F: ACGTTGGATGGTTGTGTCTGCTGCTTTCTG	70.4
	R: ACGTTGGATGTGACTGCACAGAACACATGG	
	E: CCGGCATCCCCGGCCGCTGCCCC	
c.1437G>A (479T)	F: ACGTTGGATGAGCCAAAGAAGAGGATCGTG	45.3
	R: ACGTTGGATGCCTTGGCCTCTTCTAATCTG	
	E: ATCTGTAATCAGGAGGAAT	
c.2097C>T (699G)	F: ACGTTGGATGAAGACAAGAAGCCTGCTTCC	52.0
	R: ACGTTGGATGGACACAACCGAGCTTTGAAC	
	E: GTGGAATCCTTTGCTTTCCC	

Note: F: forward primer sequence; R: reverse primer sequence; E: extended primer sequence.

**Table 3:** Associations of *CAST* variants with fatty acid composition in the longissimus thoracis muscle of Sonid sheep.

Fatty Acid Composition	c.1210C>T (R404C)			c.1437G>A in LD-M		c.2097C>T	
	Genotype			Genotype		Genotype	
	CC (253)	CT (114)	TT (11)	GG (288)	GA (84)	CC (287)	CT (89)
C10:0	0.31 ± 0.01	0.34 ± 0.02	0.35 ± 0.05	0.31 ± 0.01 ^a^	0.36 ± 0.02 ^b^	0.31 ± 0.01 ^a^	0.37 ± 0.02 ^b^
C12:0	0.44 ± 0.02 ^a^	0.59 ± 0.07 ^b^	0.52 ± 0.16 ^ab^	0.47 ± 0.03	0.56 ± 0.08	0.47 ± 0.03	0.57 ± 0.07
C14:0	1.56 ± 0.04 ^A^	1.36 ± 0.05 ^B^	1.32 ± 0.15 ^AB^	1.56 ± 0.04 ^A^	1.30 ± 0.06 ^B^	1.51 ± 0.04	1.44 ± 0.06
C18:0	9.24 ± 0.13 ^a^	8.64 ± 0.18 ^b^	8.95 ± 0.58 ^ab^	9.24 ± 0.12 ^A^	8.50 ± 0.21 ^B^	9.12 ± 0.12	8.84 ± 0.22
SFA	36.12 ± 0.35	34.79 ± 0.43	36.84 ± 1.41	36.22 ± 0.32 ^a^	34.45 ± 0.50 ^b^	35.91 ± 0.30	35.04 ± 0.59
C18:1n9t	1.71 ± 0.14	2.17 ± 0.22	1.80 ± 0.06	1.71 ± 0.12 ^a^	2.30 ± 0.27 ^b^	1.76 ± 0.12	2.28 ± 0.34
C18:1n9c	16.19 ± 0.28 ^a^	14.96 ± 0.40 ^b^	16.09 ± 0.94 ^ab^	16.14 ± 0.27 ^a^	14.77 ± 0.42 ^b^	15.99 ± 0.26	15.29 ± 0.46
C18:3n3	1.76 ± 0.04 ^a^	1.55 ± 0.08 ^b^	1.51 ± 0.24 ^ab^	1.74 ± 0.04	1.53 ± 0.09	1.75 ± 0.04 ^a^	1.53 ± 0.08 ^b^
n-6	5.45 ± 0.09 ^A^	5.47 ± 0.14 ^AB^	6.44 ± 0.40 ^B^	5.43 ± 0.08	5.61 ± 0.18	5.43 ± 0.08	5.69 ± 0.18
n-3	2.76 ± 0.06 ^A^	2.51 ± 0.09 ^B^	2.36 ± 0.30 ^B^	2.73 ± 0.06 ^A^	2.50 ± 0.10 ^B^	2.76 ± 0.06 ^a^	2.42 ± 0.09 ^b^
n-6/n-3	1.97 ± 0.23 ^A^	2.18 ± 0.59 ^B^	2.73 ± 1.22 ^B^	1.99 ± 0.24 ^A^	2.24 ± 0.71 ^B^	1.97 ± 0.28 ^A^	2.35 ± 0.56 ^B^

Note: Values are shown as the means ± standard error. Values with different superscripts within the same column differ significantly at *p* < 0.05 (a, b), *p* < 0.01 (A, B) after Bonferroni correction.

**Table 4:** Main haplotypes and their frequencies of *CAST* in Sonid sheep.

Haplotype	c.646G>C	c.1210C>T	c.1437G>A	c.2097C>T	Frequency
H1 (604)	G	C	G	C	0.798
H2 (77)	C	T	A	T	0.101
H3 (40)	G	T	G	C	0.053

**Table 5:** Associations between haplotypes of *CAST* and fatty acid composition in the longissimus thoracis muscle of Sonid sheep.

Fatty Acid Composition	Genotype of Combination (Number)		
	H1H1 (238)	H1H2 (64)	H1H3 (35)
	(GGCCGGCC)	(GCCTGACT)	(GGCTGGCC)
C12:0	0.44 ± 0.02 ^a^	0.59 ± 0.09 ^ab^	0.69 ± 0.15 ^b^
C21:0	0.54 ± 0.03 ^A^	0.52 ± 0.06 ^a^	1.2 ± 0.52 ^Bb^
C18:3n3	1.77 ± 0.05 ^a^	1.52 ± 0.11 ^b^	1.59 ± 0.14 ^ab^
n-3	2.78 ± 0.06 ^A^	2.44 ± 0.11 ^B^	2.51 ± 0.17 ^AB^
n-6/n-3	1.96 ± 0.24 ^a^	2.28 ± 0.73 ^b^	2.12 ± 0.96 ^b^

Note: Values are shown as the means ± standard error. Values with different superscripts within the same column differ significantly at *p* < 0.05 (a, b), *p* < 0.01 (A, B) after Bonferroni correction.

## Data Availability

Not applicable.

## Supplementary Materials

The following supporting information can be downloaded at https://www.mdpi.com/article/10.3390/ani13020195/s1: Table S1: Descriptive statistics for the studied traits in longissimus thoracis with the number of considered Sonid sheep, the mean value, and the standard deviation (SD); Table S2: Correlation analyses between any two traits of fatty acid compositions and classes; Table S3: Statistical difference of the *CAST* gene expression between different tissues in Sonid sheep; Table S4: Genotypic frequencies, allelic frequencies, and diversity parameters of four polymorphisms in the Sonid population; Table S5: Linkage disequilibrium as measured by D’ and *r*^2^ among four mutations in the *CAST* gene; Table S6: Associations of *CAST* variants with fatty acid composition in the longissimus thoracis muscle of Sonid sheep; Table S7: Associations between the haplotypes of *CAST* and fatty acid composition in the longissimus thoracis muscle of Sonid sheep.
