# Supplementary material for: Effects of the Expressions and Variants of the CAST Gene on the Fatty Acid Composition of the Longissimus Thoracis Muscle of Grazing Sonid Sheep
Source: Animals (Basel). 2023 Jan 4;13(2):195. doi: 10.3390/ani13020195 (PMC9855194; doi:10.3390/ani13020195)
Supplement: Supplementary file 1 [file animals-13-00195-s001.zip › animals-2068675-supplementary/Table S1. Descriptive statistics for the studied traits in longissimus thoracis with the number of considered Sonid sheep, the mean value and the standard deviation (SD).pdf]

Table S1. Descriptive statistics for the studied traits in longissimus thoracis with the number of considered Sonid sheep, the mean value, and the standard deviation (SD).

Trait	Nomenclature	N	Mean	SD
Butyric acid	C4:0	378	1.86	0.84
Caproic acid	C6:0	378	0.79	0.52
Capric acid	C10:0	378	0.32	0.13
Undecanoic acid	C11:0	378	0.46	0.19
Lauric acid	C12:0	378	0.49	0.31
Tridecanoic acid	C13:0	378	0.63	0.39
Myristic acid	C14:0	378	1.49	0.60
Pentadecanoic acid	C15:0	378	0.77	0.36
Palmitic acid	C16:0	378	16.85	3.43
Heptadecanoic acid	C17:0	378	0.70	0.88
Stearic acid	C18:0	378	9.05	1.96
Heneicosylic acid	C21:0	378	0.58	0.53
Behenic acid	C22:0	378	0.66	0.20
Tricosanoic acid	C23:0	378	0.56	0.19
Tetracosanoic acid	C24:0	378	0.53	0.22
Saturated fatty acid	SFA	378	35.74	5.24
Myristoleic acid	C14:1	378	0.72	0.94
Palmitoleic acid	C16:1	378	0.92	1.08
Ginkgolic acid	C17:1	378	0.75	0.41
Elaidic acid	C18:1n9t	378	1.85	1.44
Oleic acid	C18:1n9c	378	15.82	4.36
Eicosenoic acid	C20:1n9	378	0.70	0.18
Erucic acid	C22:1n9	378	0.57	0.25
Monounsaturated fatty acid	MUFA	378	21.34	4.89
Linoleic acid	C18:2n6c	378	4.49	1.18
α-Linolenic acid	C18:3n3	378	1.69	0.63
dihomo-γ-linolenic acid	C20:3n6	378	0.45	0.16
Arachidonic acid	C20:4n6	378	0.54	0.36
Eicosapentaenoic acid	C20:5n3	378	0.55	0.24
Docosahexaenoic acid	C22:6n3	378	0.43	0.12
Polyunsaturated fatty acid	PUFA	378	8.16	2.23
Unsaturated fatty acid	UFA	378	29.50	6.00
MUFA/SFA	MUFA/SFA	378	0.60	0.13
PUFA/SFA	PUFA/SFA	378	0.23	0.07
UFA/SFA	UFA/SFA	378	0.83	0.15
Short chain fatty acid	SCFA	378	1.86	0.84
Medium chain fatty acid	MCFA	378	2.05	0.56
Long chain fatty acid	LCFA	378	61.32	10.36
Omega 6 Polyunsaturated fatty acids	n-6	378	5.48	1.44
Omega 3 Polyunsaturated fatty acids	n-3	378	2.67	0.89
n-6/n-3	n-6/n-3	378	2.06	4.16

Essential fatty acid	EFA	378	8.16	2.23

Note: N: number of samples. Mean: mean value. SD: standard deviation.
