# Supplementary material for: Effects of the Expressions and Variants of the CAST Gene on the Fatty Acid Composition of the Longissimus Thoracis Muscle of Grazing Sonid Sheep
Source: Animals (Basel). 2023 Jan 4;13(2):195. doi: 10.3390/ani13020195 (PMC9855194; doi:10.3390/ani13020195)
Supplement: Supplementary file 1 [file animals-13-00195-s001.zip › animals-2068675-supplementary/Table S2. Correlation analyses between any two traits of fatty acid compositions and classes.pdf]

**Table S2.** Correlation analyses between any two traits of fatty acid compositions and classes.

| fatty acid | | C4:0 | C6:0 | C10:0 | C11:0 | C12:0 | C13:0 | C14:0 | C15:0 | C16:0 | C17:0 | C18:0 | C21:0 | C22:0 | C23:0 |
|---|---|---|---|---|---|---|---|---|---|---|---|---|---|---|---|
| C4:0 | *r* value | 1.000 | 0.680$^{**}$ | 0.519$^{**}$ | 0.200$^{*}$ | 0.268$^{*}$ | 0.327$^{**}$ | -0.034 | 0.197$^{*}$ | -0.284$^{**}$ | 0.001 | -0.192$^{*}$ | 0.127 | 0.053 | -0.071 |
| | *p* value | | 0.000 | 0.000 | 0.027 | 0.014 | 0.002 | 0.668 | 0.028 | 0.000 | 0.993 | 0.012 | 0.329 | 0.767 | 0.541 |
| C6:0 | *r* value | 0.680$^{**}$ | 1.000 | 0.748$^{**}$ | 0.395$^{**}$ | 0.198 | 0.433$^{**}$ | 0.146 | 0.380$^{**}$ | -0.285$^{**}$ | -0.219 | -0.046 | 0.048 | 0.027 | -0.003 |
| | *p* value | 0.000 | | 0.000 | 0.002 | 0.192 | 0.001 | 0.158 | 0.001 | 0.005 | 0.093 | 0.652 | 0.758 | 0.896 | 0.989 |
| C10:0 | *r* value | 0.519$^{**}$ | 0.748$^{**}$ | 1.000 | 0.550$^{**}$ | 0.454$^{**}$ | 0.512$^{**}$ | 0.122 | 0.319$^{**}$ | -0.221$^{*}$ | -0.144 | -0.303$^{**}$ | 0.016 | 0.061 | -0.260$^{*}$ |
| | *p* value | 0.000 | 0.000 | | 0.000 | 0.000 | 0.000 | 0.174 | 0.000 | 0.011 | 0.246 | 0.000 | 0.925 | 0.742 | 0.015 |
| C11:0 | *r* value | 0.200$^{*}$ | 0.395$^{**}$ | 0.550$^{**}$ | 1.000 | 0.144 | 0.341$^{**}$ | -0.162$^{**}$ | 0.328$^{**}$ | -0.450$^{**}$ | -0.159 | -0.321$^{**}$ | -0.043 | -0.126 | -0.387$^{**}$ |
| | *p* value | 0.027 | 0.002 | 0.000 | | 0.156 | 0.003 | 0.005 | 0.000 | 0.000 | 0.101 | 0.000 | 0.754 | 0.426 | 0.000 |
| C12:0 | *r* value | 0.268$^{*}$ | 0.198 | 0.454$^{**}$ | 0.144 | 1.000 | 0.144 | 0.057 | -0.021 | -0.016 | -0.023 | -0.300$^{**}$ | 0.504$^{**}$ | 0.397$^{*}$ | 0.003 |
| | *p* value | 0.014 | 0.192 | 0.000 | 0.156 | | 0.257 | 0.528 | 0.828 | 0.855 | 0.847 | 0.001 | 0.001 | 0.030 | 0.980 |
| C13:0 | *r* value | 0.327$^{**}$ | 0.433$^{**}$ | 0.512$^{**}$ | 0.341$^{**}$ | 0.144 | 1.000 | -0.126 | 0.243$^{*}$ | -0.126 | 0.004 | -0.052 | 0.047 | 0.088 | 0.014 |
| | *p* value | 0.002 | 0.001 | 0.000 | 0.003 | 0.257 | | 0.193 | 0.022 | 0.190 | 0.977 | 0.596 | 0.765 | 0.613 | 0.926 |
| C14:0 | *r* value | -0.034 | 0.146 | 0.122 | -0.162$^{**}$ | 0.057 | -0.126 | 1.000 | -0.072 | 0.485$^{**}$ | 0.026 | 0.567$^{**}$ | 0.074 | -0.136 | 0.234$^{**}$ |
| | *p* value | 0.668 | 0.158 | 0.174 | 0.005 | 0.528 | 0.193 | | 0.248 | 0.000 | 0.767 | 0.000 | 0.533 | 0.321 | 0.001 |
| C15:0 | *r* value | 0.197$^{*}$ | 0.380$^{**}$ | 0.319$^{**}$ | 0.328$^{**}$ | -0.021 | 0.243$^{*}$ | -0.072 | 1.000 | -0.270$^{**}$ | -0.009 | -0.217$^{**}$ | -0.025 | 0.067 | -0.338$^{**}$ |
| | *p* value | 0.028 | 0.001 | 0.000 | 0.000 | 0.828 | 0.022 | 0.248 | | 0.000 | 0.929 | 0.000 | 0.851 | 0.633 | 0.000 |
| C16:0 | *r* value | -0.284$^{**}$ | -0.285$^{**}$ | -0.221$^{*}$ | -0.450$^{**}$ | -0.016 | -0.126 | 0.485$^{**}$ | -0.270$^{**}$ | 1.000 | 0.067 | 0.590$^{**}$ | 0.025 | -0.220 | 0.498$^{**}$ |
| | *p* value | 0.000 | 0.005 | 0.011 | 0.000 | 0.855 | 0.190 | 0.000 | 0.000 | | 0.433 | 0.000 | 0.828 | 0.094 | 0.000 |
| C17:0 | *r* value | 0.001 | -0.219 | -0.144 | -0.159 | -0.023 | 0.004 | 0.026 | -0.009 | 0.067 | 1.000 | -0.016 | -0.079 | -0.105 | 0.127 |
| | *p* value | 0.993 | 0.093 | 0.246 | 0.101 | 0.847 | 0.977 | 0.767 | 0.929 | 0.433 | | 0.854 | 0.643 | 0.611 | 0.221 |
| C18:0 | *r* value | -0.192$^{*}$ | -0.046 | -0.303$^{**}$ | -0.321$^{**}$ | -0.300$^{**}$ | -0.052 | 0.567$^{**}$ | -0.217$^{**}$ | 0.590$^{**}$ | -0.016 | 1.000 | 0.041 | -0.030 | 0.387$^{**}$ |
| | *p* value | 0.012 | 0.652 | 0.000 | 0.000 | 0.001 | 0.596 | 0.000 | 0.000 | 0.000 | 0.854 | | 0.719 | 0.823 | 0.000 |
| C21:0 | *r* value | 0.127 | 0.048 | 0.016 | -0.043 | 0.504$^{**}$ | 0.047 | 0.074 | -0.025 | 0.025 | -0.079 | 0.041 | 1.000 | 0.248 | -0.107 |
| | *p* value | 0.329 | 0.758 | 0.925 | 0.754 | 0.001 | 0.765 | 0.533 | 0.851 | 0.828 | 0.643 | 0.719 | | 0.232 | 0.755 |

| | | | | | | | | | | | | | | | |
|---|---|---|---|---|---|---|---|---|---|---|---|---|---|---|---|
| C22:0 | *r* value | 0.053 | 0.027 | 0.061 | -0.126 | 0.397* | 0.088 | -0.136 | 0.067 | -0.220 | -0.105 | -0.030 | 0.248 | 1.000 | 0.160 |
| | *p* value | 0.767 | 0.896 | 0.742 | 0.426 | 0.030 | 0.613 | 0.321 | 0.633 | 0.094 | 0.611 | 0.823 | 0.232 | | 0.477 |
| C23:0 | *r* value | -0.071 | -0.003 | -0.260* | -0.387** | 0.003 | 0.014 | 0.234** | -0.338** | 0.498** | 0.127 | 0.387** | -0.107 | 0.160 | 1.000 |
| | *p* value | 0.541 | 0.989 | 0.015 | 0.000 | 0.980 | 0.926 | 0.001 | 0.000 | 0.000 | 0.221 | 0.000 | 0.755 | 0.477 | |
| C24:0 | *r* value | 0.060 | 0.061 | 0.020 | -0.109 | 0.128 | -0.197 | 0.079 | -0.174* | 0.174** | 0.047 | 0.161* | -0.053 | 0.061 | 0.292** |
| | *p* value | 0.613 | 0.737 | 0.846 | 0.111 | 0.247 | 0.175 | 0.248 | 0.023 | 0.010 | 0.657 | 0.018 | 0.857 | 0.778 | 0.000 |
| SFA | *r* value | -0.045 | 0.129 | -0.129 | -0.488** | -0.037 | 0.066 | 0.514** | -0.189** | 0.909** | 0.130 | 0.679** | 0.151 | -0.176 | 0.531** |
| | *p* value | 0.561 | 0.208 | 0.141 | 0.000 | 0.681 | 0.492 | 0.000 | 0.002 | 0.000 | 0.128 | 0.000 | 0.182 | 0.182 | 0.000 |
| | *r* value | 0.058 | 0.131 | 0.069 | 0.040 | 0.076 | 0.222* | -0.039 | -0.122 | -0.030 | -0.068 | 0.048 | -0.029 | 0.277 | 0.152 |
| C14:1 | *p* value | 0.524 | 0.238 | 0.630 | 0.718 | 0.589 | 0.049 | 0.665 | 0.254 | 0.734 | 0.565 | 0.590 | 0.820 | 0.154 | 0.369 |
| C16:1 | *r* value | -0.291** | -0.121 | -0.065 | -0.017 | -0.026 | -0.167 | 0.180** | -0.030 | 0.105 | 0.034 | 0.089 | 0.046 | 0.102 | 0.237** |
| | *p* value | 0.000 | 0.245 | 0.480 | 0.792 | 0.779 | 0.088 | 0.003 | 0.663 | 0.080 | 0.696 | 0.140 | 0.700 | 0.469 | 0.002 |
| C17:1 | *r* value | 0.078 | 0.248* | 0.297** | 0.305** | 0.341** | 0.192 | -0.095 | 0.287** | -0.171** | -0.026 | -0.104 | 0.021 | 0.363* | -0.132* |
| | *p* value | 0.368 | 0.029 | 0.002 | 0.000 | 0.000 | 0.068 | 0.114 | 0.000 | 0.003 | 0.781 | 0.079 | 0.881 | 0.027 | 0.050 |
| C18:1n9t | *r* value | -0.032 | -0.337* | -0.022 | 0.092 | -0.126 | -0.112 | -0.131 | 0.017 | -0.228** | -0.075 | -0.191* | -0.473* | -0.114 | -0.103 |
| | *p* value | 0.774 | 0.023 | 0.861 | 0.271 | 0.299 | 0.480 | 0.105 | 0.851 | 0.004 | 0.473 | 0.016 | 0.026 | 0.522 | 0.253 |
| C18:1n9c | *r* value | -0.467** | -0.388** | -0.337** | -0.421** | -0.238** | -0.249** | 0.453** | -0.315** | 0.723** | -0.003 | 0.639** | -0.142 | -0.045 | 0.451** |
| | *p* value | 0.000 | 0.000 | 0.000 | 0.000 | 0.007 | 0.009 | 0.000 | 0.000 | 0.000 | 0.972 | 0.000 | 0.209 | 0.737 | 0.000 |
| C20:1n9 | *r* value | -0.130 | -0.110 | -0.189 | -0.178 | -0.195 | -0.067 | -0.001 | -0.096 | 0.161 | 0.190 | 0.096 | 0.599 | -0.015 | -0.014 |
| | *p* value | 0.308 | 0.510 | 0.336 | 0.080 | 0.329 | 0.741 | 0.990 | 0.441 | 0.093 | 0.133 | 0.321 | 0.067 | 0.972 | 0.891 |
| C22:1n9 | *r* value | 0.177 | 0.153 | 0.144 | -0.047 | 0.215 | 0.239* | -0.024 | 0.161 | -0.123 | 0.145 | -0.048 | 0.083 | 0.117 | 0.280 |
| | *p* value | 0.070 | 0.208 | 0.401 | 0.720 | 0.160 | 0.050 | 0.808 | 0.187 | 0.211 | 0.320 | 0.625 | 0.530 | 0.560 | 0.354 |
| MUFA | *r* value | -0.413** | -0.367** | -0.251** | -0.448** | -0.181* | -0.135 | 0.306** | -0.290** | 0.610** | -0.007 | 0.446** | -0.140 | -0.054 | 0.381** |
| | *p* value | 0.000 | 0.000 | 0.004 | 0.000 | 0.042 | 0.158 | 0.000 | 0.000 | 0.000 | 0.940 | 0.000 | 0.217 | 0.683 | 0.000 |
| C18:2n6c | *r* value | -0.182* | -0.219* | -0.207* | -0.383** | -0.100 | -0.291** | 0.209** | -0.188** | 0.579** | 0.002 | 0.362** | -0.089 | 0.022 | 0.498** |
| | *p* value | 0.017 | 0.031 | 0.023 | 0.000 | 0.268 | 0.002 | 0.000 | 0.003 | 0.000 | 0.984 | 0.000 | 0.443 | 0.875 | 0.000 |

| | | | | | | | | | | | | | | | |
|---|---|---|---|---|---|---|---|---|---|---|---|---|---|---|---|
| C18:3n3 | *r* value | -0.138 | -0.091 | -0.247* | -0.261** | -0.177 | -0.040 | 0.223** | -0.096 | 0.299** | 0.045 | 0.206** | -0.126 | -0.084 | 0.369** |
| | *p* value | 0.137 | 0.470 | 0.010 | 0.000 | 0.079 | 0.697 | 0.000 | 0.173 | 0.000 | 0.684 | 0.001 | 0.318 | 0.565 | 0.000 |
| C20:3n6 | *r* value | 0.210 | -0.134 | 0.053 | -0.040 | -0.126 | -0.103 | -0.062 | 0.140 | 0.287** | 0.026 | 0.002 | 0.198 | -0.222 | 0.082 |
| | *p* value | 0.061 | 0.340 | 0.718 | 0.718 | 0.398 | 0.475 | 0.560 | 0.201 | 0.005 | 0.842 | 0.981 | 0.246 | 0.285 | 0.530 |
| C20:4n6 | *r* value | -0.017 | 0.064 | 0.089 | -0.081 | 0.165 | 0.042 | 0.251** | -0.164 | 0.219** | -0.002 | 0.218** | 0.212 | -0.094 | -0.002 |
| | *p* value | 0.856 | 0.602 | 0.483 | 0.367 | 0.183 | 0.712 | 0.001 | 0.063 | 0.004 | 0.983 | 0.005 | 0.085 | 0.614 | 0.988 |
| C20:5n3 | *r* value | -0.067 | -0.136 | -0.017 | -0.357** | 0.126 | -0.003 | -0.048 | -0.139 | 0.064 | 0.159 | -0.064 | -0.249* | -0.287 | -0.383 |
| | *r* value | 0.502 | 0.265 | 0.917 | 0.001 | 0.415 | 0.979 | 0.590 | 0.183 | 0.465 | 0.254 | 0.468 | 0.037 | 0.095 | 0.245 |
| C22:6n3 | *p* value | 0.088 | -0.089 | -0.231 | -0.252* | -0.176 | 0.456* | 0.414** | -0.259* | 0.446** | 0.000 | 0.371** | -0.193 | -0.111 | 0.347** |
| | *r* value | 0.572 | 0.671 | 0.146 | 0.050 | 0.236 | 0.011 | 0.001 | 0.044 | 0.000 | 0.997 | 0.003 | 0.679 | 0.681 | 0.006 |
| PUFA | *p* value | -0.273** | -0.045 | -0.104 | -0.276** | -0.161 | -0.135 | 0.139** | 0.013 | 0.424** | 0.000 | 0.116* | -0.182 | -0.020 | 0.291** |
| | *r* value | 0.000 | 0.663 | 0.239 | 0.000 | 0.072 | 0.160 | 0.009 | 0.829 | 0.000 | 1.000 | 0.026 | 0.110 | 0.879 | 0.000 |
| UFA | *p* value | -0.456** | -0.343** | -0.242** | -0.453** | -0.212* | -0.169 | 0.299** | -0.225** | 0.650** | -0.005 | 0.397** | -0.193 | -0.076 | 0.408** |
| | *r* value | 0.000 | 0.001 | 0.005 | 0.000 | 0.017 | 0.077 | 0.000 | 0.000 | 0.000 | 0.952 | 0.000 | 0.087 | 0.566 | 0.000 |
| MUFA/SFA | *p* value | -0.409** | -0.437** | -0.195* | -0.172** | -0.194* | -0.193* | -0.039 | -0.205** | 0.033 | -0.114 | -0.012 | -0.227* | 0.016 | -0.008 |
| | *r* value | 0.000 | 0.000 | 0.025 | 0.002 | 0.028 | 0.043 | 0.465 | 0.001 | 0.520 | 0.183 | 0.811 | 0.043 | 0.902 | 0.899 |
| PUFA/SFA | *p* value | -0.288** | -0.105 | -0.067 | -0.060 | -0.166 | -0.169 | -0.110* | 0.108 | 0.008 | -0.070 | -0.193** | -0.254* | 0.067 | -0.067 |
| | *r* value | 0.000 | 0.306 | 0.447 | 0.282 | 0.063 | 0.077 | 0.039 | 0.076 | 0.876 | 0.413 | 0.000 | 0.025 | 0.615 | 0.307 |
| UFA/SFA | *p* value | -0.487** | -0.438** | -0.194* | -0.161** | -0.243** | -0.249** | -0.087 | -0.119 | 0.027 | -0.135 | -0.105* | -0.285* | 0.029 | -0.033 |
| | *p* value | 0.000 | 0.000 | 0.026 | 0.003 | 0.006 | 0.009 | 0.106 | 0.051 | 0.606 | 0.115 | 0.042 | 0.010 | 0.826 | 0.617 |
| SCFA | *r* value | 1.000** | 0.680** | 0.519** | 0.200* | 0.268* | 0.327** | -0.034 | 0.197* | -0.284** | 0.001 | -0.192* | 0.127 | 0.053 | -0.071 |
| | *p* value | 0.000 | 0.000 | 0.000 | 0.027 | 0.014 | 0.002 | 0.668 | 0.028 | 0.000 | 0.993 | 0.012 | 0.329 | 0.767 | 0.541 |
| MCFA | *r* value | 0.261** | 0.730** | 0.695** | 0.286** | 0.572** | 0.326** | -0.139** | 0.229** | -0.185** | -0.083 | -0.384** | -0.034 | 0.140 | -0.149* |
| | *p* value | 0.001 | 0.000 | 0.000 | 0.000 | 0.000 | 0.001 | 0.009 | 0.000 | 0.000 | 0.339 | 0.000 | 0.765 | 0.289 | 0.024 |
| LCFA | *r* value | -0.418** | -0.308** | -0.278** | -0.520** | -0.204* | -0.121 | 0.483** | -0.250** | 0.855** | 0.062 | 0.646** | -0.053 | -0.140 | 0.500** |
| | *p* value | 0.000 | 0.002 | 0.001 | 0.000 | 0.021 | 0.209 | 0.000 | 0.000 | 0.000 | 0.469 | 0.000 | 0.644 | 0.289 | 0.000 |

| | | | | | | | | | | | | | | | |
|---|---|---|---|---|---|---|---|---|---|---|---|---|---|---|---|
| n-6 | *r* value | -0.240** | -0.216* | -0.150 | -0.325** | -0.057 | -0.234* | 0.136* | -0.152* | 0.520** | 0.010 | 0.215** | -0.080 | 0.051 | 0.452** |
| | *p* value | 0.008 | 0.010 | 0.010 | 0.000 | 0.223 | 0.004 | 0.001 | 0.003 | 0.000 | 0.764 | 0.000 | 0.394 | 0.945 | 0.000 |
| n-3 | *r* value | -0.021 | 0.183 | -0.045 | -0.050 | -0.182 | 0.096 | 0.332** | -0.003 | 0.320** | -0.022 | 0.189** | -0.199 | -0.011 | 0.199* |
| | *p* value | 0.137 | 0.470 | 0.010 | 0.000 | 0.079 | 0.697 | 0.000 | 0.173 | 0.000 | 0.684 | 0.001 | 0.318 | 0.565 | 0.000 |
| n-6/n-3 | *r* value | -0.139 | -0.327** | -0.211* | -0.289** | 0.158 | -0.226* | -0.349** | -0.168* | -0.022 | 0.030 | -0.145* | 0.223 | 0.104 | 0.132 |
| | *p* value | 0.377 | 0.105 | 0.461 | 0.963 | 0.213 | 0.168 | 0.005 | 0.739 | 0.095 | 0.894 | 0.191 | 0.108 | 0.236 | 0.005 |
| EFA | *r* value | -0.273** | -0.045 | -0.104 | -0.276** | -0.161 | -0.135 | 0.139** | 0.013 | 0.424** | 0.000 | 0.116* | -0.182 | -0.020 | 0.291** |
| | *p* value | 0.002 | 0.510 | 0.143 | 0.000 | 0.013 | 0.056 | 0.000 | 0.981 | 0.000 | 0.978 | 0.000 | 0.130 | 0.758 | 0.000 |

Note: *$p < 0.05$, **$p < 0.01$.

**Table S2.** (continued)

| fatty acid | | C24:0 | SFA | C14:1 | C16:1 | C17:1 | C18:1 n9t | C18:1 n9c | C20:1 n9 | C22:1 n9 | MUFA | C18:2 n6c | C18:3 n3 | C20:3 n6 | C20:4 n6 |
|---|---|---|---|---|---|---|---|---|---|---|---|---|---|---|---|
| C4:0 | *r* value | 0.060 | -0.045 | 0.058 | -0.291** | 0.078 | -0.032 | -0.467** | -0.130 | 0.177 | -0.413** | -0.182* | -0.138 | 0.210 | -0.017 |
| | *p* value | 0.613 | 0.561 | 0.524 | 0.000 | 0.368 | 0.774 | 0.000 | 0.308 | 0.070 | 0.000 | 0.017 | 0.137 | 0.061 | 0.856 |
| C6:0 | *r* value | 0.061 | 0.129 | 0.131 | -0.121 | 0.248* | -0.337* | -0.388** | -0.110 | 0.153 | -0.367** | -0.219* | -0.091 | -0.134 | 0.064 |
| | *p* value | 0.737 | 0.208 | 0.238 | 0.245 | 0.029 | 0.023 | 0.000 | 0.510 | 0.208 | 0.000 | 0.031 | 0.470 | 0.340 | 0.602 |
| C10:0 | *r* value | 0.020 | -0.129 | 0.069 | -0.065 | 0.297** | -0.022 | -0.337** | -0.189 | 0.144 | -0.251** | -0.207* | -0.247* | 0.053 | 0.089 |
| | *p* value | 0.846 | 0.141 | 0.630 | 0.480 | 0.002 | 0.861 | 0.000 | 0.336 | 0.401 | 0.004 | 0.023 | 0.010 | 0.718 | 0.483 |
| C11:0 | *r* value | -0.109 | -0.488** | 0.040 | -0.017 | 0.305** | 0.092 | -0.421** | -0.178 | -0.047 | -0.448** | -0.383** | -0.261** | -0.040 | -0.081 |
| | *p* value | 0.111 | 0.000 | 0.718 | 0.792 | 0.000 | 0.271 | 0.000 | 0.080 | 0.720 | 0.000 | 0.000 | 0.000 | 0.718 | 0.367 |
| C12:0 | *r* value | 0.128 | -0.037 | 0.076 | -0.026 | 0.341** | -0.126 | -0.238** | -0.195 | 0.215 | -0.181* | -0.100 | -0.177 | -0.126 | 0.165 |
| | *p* value | 0.247 | 0.681 | 0.589 | 0.779 | 0.000 | 0.299 | 0.007 | 0.329 | 0.160 | 0.042 | 0.268 | 0.079 | 0.398 | 0.183 |
| C13:0 | *r* value | -0.197 | 0.066 | 0.222* | -0.167 | 0.192 | -0.112 | -0.249** | -0.067 | 0.239* | -0.135 | -0.291** | -0.040 | -0.103 | 0.042 |
| | *p* value | 0.175 | 0.492 | 0.049 | 0.088 | 0.068 | 0.480 | 0.009 | 0.741 | 0.050 | 0.158 | 0.002 | 0.697 | 0.475 | 0.712 |
| C14:0 | *r* value | 0.079 | 0.514** | -0.039 | 0.180** | -0.095 | -0.131 | 0.453** | -0.001 | -0.024 | 0.306** | 0.209** | 0.223** | -0.062 | 0.251** |
| | *p* value | 0.248 | 0.000 | 0.665 | 0.003 | 0.114 | 0.105 | 0.000 | 0.990 | 0.808 | 0.000 | 0.000 | 0.000 | 0.560 | 0.001 |
| C15:0 | *r* value | -0.174* | -0.189** | -0.122 | -0.030 | 0.287** | 0.017 | -0.315** | -0.096 | 0.161 | -0.290** | -0.188** | -0.096 | 0.140 | -0.164 |
| | *p* value | 0.023 | 0.002 | 0.254 | 0.663 | 0.000 | 0.851 | 0.000 | 0.441 | 0.187 | 0.000 | 0.003 | 0.173 | 0.201 | 0.063 |
| C16:0 | *r* value | 0.174** | 0.909** | -0.030 | 0.105 | -0.171** | -0.228** | 0.723** | 0.161 | -0.123 | 0.610** | 0.579** | 0.299** | 0.287** | 0.219** |
| | *p* value | 0.010 | 0.000 | 0.734 | 0.080 | 0.003 | 0.004 | 0.000 | 0.093 | 0.211 | 0.000 | 0.000 | 0.000 | 0.005 | 0.004 |
| C17:0 | *r* value | 0.047 | 0.130 | -0.068 | 0.034 | -0.026 | -0.075 | -0.003 | 0.190 | 0.145 | -0.007 | 0.002 | 0.045 | 0.026 | -0.002 |
| | *p* value | 0.657 | 0.128 | 0.565 | 0.696 | 0.781 | 0.473 | 0.972 | 0.133 | 0.320 | 0.940 | 0.984 | 0.684 | 0.842 | 0.983 |
| C18:0 | *r* value | 0.161* | 0.679** | 0.048 | 0.089 | -0.104 | -0.191* | 0.639** | 0.096 | -0.048 | 0.446** | 0.362** | 0.206** | 0.002 | 0.218** |
| | *p* value | 0.018 | 0.000 | 0.590 | 0.140 | 0.079 | 0.016 | 0.000 | 0.321 | 0.625 | 0.000 | 0.000 | 0.001 | 0.981 | 0.005 |
| C21:0 | *r* value | -0.053 | 0.151 | -0.029 | 0.046 | 0.021 | -0.473* | -0.142 | 0.599 | 0.083 | -0.140 | -0.089 | -0.126 | 0.198 | 0.212 |

| | | | | | | | | | | | | | | | |
|---|---|---|---|---|---|---|---|---|---|---|---|---|---|---|---|
| | *p* value | 0.857 | 0.182 | 0.820 | 0.700 | 0.881 | 0.026 | 0.209 | 0.067 | 0.530 | 0.217 | 0.443 | 0.318 | 0.246 | 0.085 |
| C22:0 | *r* value | 0.061 | -0.176 | 0.277 | 0.102 | 0.363* | -0.114 | -0.045 | -0.015 | 0.117 | -0.054 | 0.022 | -0.084 | -0.222 | -0.094 |
| | *p* value | 0.778 | 0.182 | 0.154 | 0.469 | 0.027 | 0.522 | 0.737 | 0.972 | 0.560 | 0.683 | 0.875 | 0.565 | 0.285 | 0.614 |
| C23:0 | *r* value | 0.292** | 0.531** | 0.152 | 0.237** | -0.132* | -0.103 | 0.451** | -0.014 | 0.280 | 0.381** | 0.498** | 0.369** | 0.082 | -0.002 |
| | *p* value | 0.000 | 0.000 | 0.369 | 0.002 | 0.050 | 0.253 | 0.000 | 0.891 | 0.354 | 0.000 | 0.000 | 0.000 | 0.530 | 0.988 |
| C24:0 | *r* value | 1.000 | 0.247** | 0.042 | -0.059 | 0.073 | -0.103 | 0.164* | -0.050 | 0.385 | 0.115 | 0.343** | 0.086 | -0.059 | 0.015 |
| | *p* value | | 0.000 | 0.801 | 0.451 | 0.300 | 0.259 | 0.015 | 0.646 | 0.174 | 0.088 | 0.000 | 0.328 | 0.655 | 0.921 |
| SFA | *r* value | 0.247** | 1.000 | -0.043 | 0.091 | -0.205** | -0.283** | 0.724** | 0.151 | -0.011 | 0.624** | 0.568** | 0.299** | 0.190 | 0.190* |
| | *p* value | 0.000 | | 0.629 | 0.128 | 0.000 | 0.000 | 0.000 | 0.116 | 0.914 | 0.000 | 0.000 | 0.000 | 0.067 | 0.013 |
| | *r* value | 0.042 | -0.043 | 1.000 | 0.016 | -0.014 | 0.018 | 0.074 | 0.099 | 0.053 | 0.261** | 0.025 | -0.083 | -0.055 | 0.134 |
| C14:1 | *p* value | 0.801 | 0.629 | | 0.858 | 0.893 | 0.908 | 0.403 | 0.560 | 0.595 | 0.003 | 0.777 | 0.394 | 0.673 | 0.163 |
| C16:1 | *r* value | -0.059 | 0.091 | 0.016 | 1.000 | -0.205** | -0.056 | 0.209** | -0.005 | -0.107 | 0.392** | 0.061 | 0.028 | -0.143 | 0.128 |
| | *p* value | 0.451 | 0.128 | 0.858 | | 0.002 | 0.499 | 0.000 | 0.961 | 0.287 | 0.000 | 0.317 | 0.699 | 0.171 | 0.124 |
| C17:1 | *r* value | 0.073 | -0.205** | -0.014 | -0.205** | 1.000 | 0.000 | -0.258** | -0.071 | -0.055 | -0.166** | -0.251** | -0.167* | -0.100 | 0.090 |
| | *p* value | 0.300 | 0.000 | 0.893 | 0.002 | | 0.997 | 0.000 | 0.485 | 0.646 | 0.004 | 0.000 | 0.023 | 0.358 | 0.361 |
| C18:1n9t | *r* value | -0.103 | -0.283** | 0.018 | -0.056 | 0.000 | 1.000 | -0.133 | 0.155 | 0.104 | 0.148 | -0.065 | -0.006 | -0.151 | 0.015 |
| | *p* value | 0.259 | 0.000 | 0.908 | 0.499 | 0.997 | | 0.096 | 0.201 | 0.599 | 0.062 | 0.418 | 0.953 | 0.262 | 0.914 |
| C18:1n9c | *r* value | 0.164* | 0.724** | 0.074 | 0.209** | -0.258** | -0.133 | 1.000 | 0.107 | -0.118 | 0.909** | 0.484** | 0.266** | -0.131 | 0.274** |
| | *p* value | 0.015 | 0.000 | 0.403 | 0.000 | 0.000 | 0.096 | | 0.270 | 0.230 | 0.000 | 0.000 | 0.000 | 0.212 | 0.000 |
| C20:1n9 | *r* value | -0.050 | 0.151 | 0.099 | -0.005 | -0.071 | 0.155 | 0.107 | 1.000 | -0.164 | 0.213* | 0.369** | 0.243 | 0.285 | 0.075 |
| | *p* value | 0.646 | 0.116 | 0.560 | 0.961 | 0.485 | 0.201 | 0.270 | | 0.444 | 0.026 | 0.000 | 0.316 | 0.067 | 0.728 |
| C22:1n9 | *r* value | 0.385 | -0.011 | 0.053 | -0.107 | -0.055 | 0.104 | -0.118 | -0.164 | 1.000 | 0.012 | -0.142 | -0.144 | 0.016 | 0.109 |
| | *p* value | 0.174 | 0.914 | 0.595 | 0.287 | 0.646 | 0.599 | 0.230 | 0.444 | | 0.906 | 0.147 | 0.161 | 0.921 | 0.278 |
| MUFA | *r* value | 0.115 | 0.624** | 0.261** | 0.392** | -0.166** | 0.148 | 0.909** | 0.213* | 0.012 | 1.000 | 0.417** | 0.219** | -0.159 | 0.250** |
| | *p* value | 0.088 | 0.000 | 0.003 | 0.000 | 0.004 | 0.062 | 0.000 | 0.026 | 0.906 | | 0.000 | 0.000 | 0.126 | 0.001 |
| C18:2n6c | *r* value | 0.343** | 0.568** | 0.025 | 0.061 | -0.251** | -0.065 | 0.484** | 0.369** | -0.142 | 0.417** | 1.000 | 0.227** | 0.037 | 0.338** |

| | | | | | | | | | | | | | | | |
|---|---|---|---|---|---|---|---|---|---|---|---|---|---|---|---|
| | *p* value | 0.000 | 0.000 | 0.777 | 0.317 | 0.000 | 0.418 | 0.000 | 0.000 | 0.147 | 0.000 | | 0.000 | 0.725 | 0.000 |
| C18:3n3 | *r* value | 0.086 | 0.299** | -0.083 | 0.028 | -0.167* | -0.006 | 0.266** | 0.243 | -0.144 | 0.219** | 0.227** | 1.000 | -0.046 | 0.050 |
| | *p* value | 0.328 | 0.000 | 0.394 | 0.699 | 0.023 | 0.953 | 0.000 | 0.316 | 0.161 | 0.000 | 0.000 | | 0.733 | 0.541 |
| C20:3n6 | *r* value | -0.059 | 0.190 | -0.055 | -0.143 | -0.100 | -0.151 | -0.131 | 0.285 | 0.016 | -0.159 | 0.037 | -0.046 | 1.000 | -0.207 |
| | *p* value | 0.655 | 0.067 | 0.673 | 0.171 | 0.358 | 0.262 | 0.212 | 0.067 | 0.921 | 0.126 | 0.725 | 0.733 | | 0.163 |
| C20:4n6 | *r* value | 0.015 | 0.190* | 0.134 | 0.128 | 0.090 | 0.015 | 0.274** | 0.075 | 0.109 | 0.250** | 0.338** | 0.050 | -0.207 | 1.000 |
| | *p* value | 0.921 | 0.013 | 0.163 | 0.124 | 0.361 | 0.914 | 0.000 | 0.728 | 0.278 | 0.001 | 0.000 | 0.541 | 0.163 | |
| C20:5n3 | *r* value | -0.327 | -0.055 | 0.115 | -0.062 | -0.038 | -0.137 | -0.029 | 0.617** | 0.210* | 0.019 | 0.099 | 0.104 | 0.071 | 0.132 |
| | *p* value | 0.300 | 0.533 | 0.245 | 0.507 | 0.749 | 0.400 | 0.746 | 0.003 | 0.035 | 0.833 | 0.260 | 0.262 | 0.664 | 0.143 |
| C22:6n3 | *r* value | 0.180 | 0.449** | 0.416* | 0.053 | -0.147 | -0.154 | 0.309* | 0.515** | 0.363 | 0.000 | 0.249* | 0.183 | 0.238 | -0.083 |
| | *p* value | 0.165 | 0.000 | 0.035 | 0.677 | 0.251 | 0.295 | 0.013 | 0.004 | 0.377 | 0.999 | 0.047 | 0.265 | 0.096 | 0.742 |
| PUFA | *r* value | 0.036 | 0.422** | 0.014 | 0.119* | -0.261** | -0.156 | 0.354** | 0.377** | -0.095 | 0.331** | 0.775** | 0.461** | 0.047 | 0.440** |
| | *p* value | 0.598 | 0.000 | 0.875 | 0.048 | 0.000 | 0.050 | 0.000 | 0.000 | 0.334 | 0.000 | 0.000 | 0.000 | 0.653 | 0.000 |
| UFA | *r* value | 0.110 | 0.660** | 0.240** | 0.376** | -0.229** | 0.071 | 0.854** | 0.333** | -0.022 | 0.933** | 0.605** | 0.345** | -0.121 | 0.350** |
| | *p* value | 0.102 | 0.000 | 0.006 | 0.000 | 0.000 | 0.375 | 0.000 | 0.000 | 0.823 | 0.000 | 0.000 | 0.000 | 0.246 | 0.000 |
| MUFA/SF | *r* value | -0.084 | -0.011 | 0.311** | 0.371** | -0.031 | 0.508** | 0.547** | 0.097 | 0.013 | 0.757** | 0.074 | 0.037 | -0.286** | 0.182* |
| A | *p* value | 0.216 | 0.833 | 0.000 | 0.000 | 0.600 | 0.000 | 0.000 | 0.315 | 0.895 | 0.000 | 0.165 | 0.553 | 0.005 | 0.017 |
| PUFA/SF | *r* value | -0.134* | -0.045 | 0.040 | 0.078 | -0.176** | 0.035 | 0.021 | 0.346** | -0.096 | 0.054 | 0.535** | 0.360** | -0.041 | 0.374** |
| A | *p* value | 0.047 | 0.385 | 0.650 | 0.192 | 0.003 | 0.663 | 0.688 | 0.000 | 0.329 | 0.295 | 0.000 | 0.000 | 0.695 | 0.000 |
| UFA/SFA | *r* value | -0.129 | -0.035 | 0.296** | 0.359** | -0.103 | 0.465** | 0.455** | 0.242* | -0.022 | 0.660** | 0.274** | 0.180** | -0.267** | 0.290** |
| | *p* value | 0.056 | 0.503 | 0.001 | 0.000 | 0.079 | 0.000 | 0.000 | 0.011 | 0.824 | 0.000 | 0.000 | 0.004 | 0.009 | 0.000 |
| SCFA | *r* value | 0.060 | -0.045 | 0.058 | -0.291** | 0.078 | -0.032 | -0.467** | -0.130 | 0.177 | -0.413** | -0.182* | -0.138 | 0.210 | -0.017 |
| | *p* value | 0.613 | 0.561 | 0.524 | 0.000 | 0.368 | 0.774 | 0.000 | 0.308 | 0.070 | 0.000 | 0.017 | 0.137 | 0.061 | 0.856 |
| MCFA | *r* value | 0.012 | -0.011 | -0.054 | -0.074 | 0.074 | -0.042 | -0.238** | -0.101 | 0.043 | -0.138** | -0.108* | -0.097 | -0.276** | -0.161* |
| | *p* value | 0.857 | 0.825 | 0.544 | 0.218 | 0.210 | 0.605 | 0.000 | 0.297 | 0.670 | 0.007 | 0.042 | 0.121 | 0.007 | 0.037 |
| LCFA | *r* value | 0.190** | 0.883** | 0.131 | 0.277** | -0.230** | -0.091 | 0.891** | 0.278** | -0.047 | 0.874** | 0.638** | 0.365** | 0.028 | 0.338** |

| | | | | | | | | | | | | | | | |
|---|---|---|---|---|---|---|---|---|---|---|---|---|---|---|---|
| | *p* value | 0.005 | 0.000 | 0.136 | 0.000 | 0.000 | 0.253 | 0.000 | 0.003 | 0.635 | 0.000 | 0.000 | 0.000 | 0.788 | 0.000 |
| n-6 | *r* value | 0.296** | 0.494** | 0.033 | 0.100 | -0.236** | -0.071 | 0.443** | 0.421** | -0.109 | 0.416** | 0.955** | 0.191** | 0.173 | 0.509** |
| | *p* value | 0.000 | 0.000 | 0.728 | 0.393 | 0.000 | 0.434 | 0.000 | 0.000 | 0.195 | 0.000 | 0.000 | 0.001 | 0.266 | 0.000 |
| n-3 | *r* value | 0.019 | 0.332** | -0.016 | 0.075 | -0.255** | -0.128 | 0.280** | 0.115 | -0.025 | 0.188** | 0.243** | 0.899** | -0.031 | 0.153* |
| | *p* value | 0.328 | 0.000 | 0.394 | 0.699 | 0.023 | 0.953 | 0.000 | 0.316 | 0.161 | 0.000 | 0.000 | 0.000 | 0.733 | 0.541 |
| n-6/n-3 | *r* value | 0.104 | -0.044 | 0.003 | -0.051 | 0.082 | 0.117 | -0.035 | -0.150 | 0.000 | 0.078 | 0.159** | -0.624** | 0.010 | 0.078 |
| | *p* value | 0.204 | 0.340 | 0.266 | 0.966 | 0.183 | 0.212 | 0.447 | 0.947 | 0.461 | 0.254 | 0.000 | 0.000 | 0.270 | 0.050 |
| EFA | *r* value | 0.036 | 0.422** | 0.014 | 0.119* | -0.261** | -0.156 | 0.354** | 0.377** | -0.095 | 0.331** | 0.775** | 0.461** | 0.047 | 0.440** |
| | *p* value | 0.433 | 0.000 | 0.929 | 0.119 | 0.000 | 0.063 | 0.000 | 0.000 | 0.105 | 0.000 | 0.000 | 0.000 | 0.941 | 0.001 |

Note: *$p < 0.05$, **$p < 0.01$.

**Table S2.** (continued)

| fatty acid | | C20:5 n3 | C22:6 n3 | PUFA | UFA | MUFA/SFA | PUFA/SFA | UFA/SFA | SCFA | MCFA | LCFA | n-6 | n-3 | n-6/n-3 | EFA |
|---|---|---|---|---|---|---|---|---|---|---|---|---|---|---|---|
| C4:0 | *r* value | -0.067 | 0.088 | -0.273** | -0.456** | -0.409** | -0.288** | -0.487** | 1.000** | 0.261** | -0.418** | -0.240** | -0.021 | -0.139 | -0.273** |
| | *p* value | 0.502 | 0.572 | 0.000 | 0.000 | 0.000 | 0.000 | 0.000 | 0.000 | 0.001 | 0.000 | 0.008 | 0.137 | 0.377 | 0.002 |
| C6:0 | *r* value | -0.136 | -0.089 | -0.045 | -0.343** | -0.437** | -0.105 | -0.438** | 0.680** | 0.730** | -0.308** | -0.216* | 0.183 | -0.327** | -0.045 |
| | *p* value | 0.265 | 0.671 | 0.663 | 0.001 | 0.000 | 0.306 | 0.000 | 0.000 | 0.000 | 0.002 | 0.010 | 0.470 | 0.105 | 0.510 |
| C10:0 | *r* value | -0.017 | -0.231 | -0.104 | -0.242** | -0.195* | -0.067 | -0.194* | 0.519** | 0.695** | -0.278** | -0.150 | -0.045 | -0.211* | -0.104 |
| | *p* value | 0.917 | 0.146 | 0.239 | 0.005 | 0.025 | 0.447 | 0.026 | 0.000 | 0.000 | 0.001 | 0.010 | 0.010 | 0.461 | 0.143 |
| C11:0 | *r* value | -0.357** | -0.252* | -0.276** | -0.453** | -0.172** | -0.060 | -0.161** | 0.200* | 0.286** | -0.520** | -0.325** | -0.050 | -0.289** | -0.276** |
| | *p* value | 0.001 | 0.050 | 0.000 | 0.000 | 0.002 | 0.282 | 0.003 | 0.027 | 0.000 | 0.000 | 0.000 | 0.000 | 0.963 | 0.000 |
| C12:0 | *r* value | 0.126 | -0.176 | -0.161 | -0.212* | -0.194* | -0.166 | -0.243** | 0.268* | 0.572** | -0.204* | -0.057 | -0.182 | 0.158 | -0.161 |
| | *p* value | 0.415 | 0.236 | 0.072 | 0.017 | 0.028 | 0.063 | 0.006 | 0.014 | 0.000 | 0.021 | 0.223 | 0.079 | 0.213 | 0.013 |
| C13:0 | *r* value | -0.003 | 0.456* | -0.135 | -0.169 | -0.193* | -0.169 | -0.249** | 0.327** | 0.326** | -0.121 | -0.234* | 0.096 | -0.226* | -0.135 |
| | *p* value | 0.979 | 0.011 | 0.160 | 0.077 | 0.043 | 0.077 | 0.009 | 0.002 | 0.001 | 0.209 | 0.004 | 0.697 | 0.168 | 0.056 |
| C14:0 | *r* value | -0.048 | 0.414** | 0.139** | 0.299** | -0.039 | -0.110* | -0.087 | -0.034 | -0.139** | 0.483** | 0.136* | 0.332** | -0.349** | 0.139** |
| | *p* value | 0.590 | 0.001 | 0.009 | 0.000 | 0.465 | 0.039 | 0.106 | 0.668 | 0.009 | 0.000 | 0.001 | 0.000 | 0.005 | 0.000 |
| C15:0 | *r* value | -0.139 | -0.259* | 0.013 | -0.225** | -0.205** | 0.108 | -0.119 | 0.197* | 0.229** | -0.250** | -0.152* | -0.003 | -0.168* | 0.013 |
| | *p* value | 0.183 | 0.044 | 0.829 | 0.000 | 0.001 | 0.076 | 0.051 | 0.028 | 0.000 | 0.000 | 0.003 | 0.173 | 0.739 | 0.981 |
| C16:0 | *r* value | 0.064 | 0.446** | 0.424** | 0.650** | 0.033 | 0.008 | 0.027 | -0.284** | -0.185** | 0.855** | 0.520** | 0.320** | -0.022 | 0.424** |
| | *p* value | 0.465 | 0.000 | 0.000 | 0.000 | 0.520 | 0.876 | 0.606 | 0.000 | 0.000 | 0.000 | 0.000 | 0.000 | 0.095 | 0.000 |
| C17:0 | *r* value | 0.159 | 0.000 | 0.000 | -0.005 | -0.114 | -0.070 | -0.135 | 0.001 | -0.083 | 0.062 | 0.010 | -0.022 | 0.030 | 0.000 |
| | *p* value | 0.254 | 0.997 | 1.000 | 0.952 | 0.183 | 0.413 | 0.115 | 0.993 | 0.339 | 0.469 | 0.764 | 0.684 | 0.894 | 0.978 |
| C18:0 | *r* value | -0.064 | 0.371** | 0.116* | 0.397** | -0.012 | -0.193** | -0.105* | -0.192* | -0.384** | 0.646** | 0.215** | 0.189** | -0.145* | 0.116* |
| | *p* value | 0.468 | 0.003 | 0.026 | 0.000 | 0.811 | 0.000 | 0.042 | 0.012 | 0.000 | 0.000 | 0.000 | 0.001 | 0.191 | 0.000 |
| C21:0 | *r* value | -0.249* | -0.193 | -0.182 | -0.193 | -0.227* | -0.254* | -0.285* | 0.127 | -0.034 | -0.053 | -0.080 | -0.199 | 0.223 | -0.182 |

| | | | | | | | | | | | | | | | |
|---|---|---|---|---|---|---|---|---|---|---|---|---|---|---|---|
| | *p* value | 0.037 | 0.679 | 0.110 | 0.087 | 0.043 | 0.025 | 0.010 | 0.329 | 0.765 | 0.644 | 0.394 | 0.318 | 0.108 | 0.130 |
| C22:0 | *r* value | -0.287 | -0.111 | -0.020 | -0.076 | 0.016 | 0.067 | 0.029 | 0.053 | 0.140 | -0.140 | 0.051 | -0.011 | 0.104 | -0.020 |
| | *p* value | 0.095 | 0.681 | 0.879 | 0.566 | 0.902 | 0.615 | 0.826 | 0.767 | 0.289 | 0.289 | 0.945 | 0.565 | 0.236 | 0.758 |
| C23:0 | *r* value | -0.383 | 0.347** | 0.291** | 0.408** | -0.008 | -0.067 | -0.033 | -0.071 | -0.149* | 0.500** | 0.452** | 0.199* | 0.132 | 0.291** |
| | *p* value | 0.245 | 0.006 | 0.000 | 0.000 | 0.899 | 0.307 | 0.617 | 0.541 | 0.024 | 0.000 | 0.000 | 0.000 | 0.005 | 0.000 |
| C24:0 | *r* value | -0.327 | 0.180 | 0.036 | 0.110 | -0.084 | -0.134* | -0.129 | 0.060 | 0.012 | 0.190** | 0.296** | 0.019 | 0.104 | 0.036 |
| | *p* value | 0.300 | 0.165 | 0.598 | 0.102 | 0.216 | 0.047 | 0.056 | 0.613 | 0.857 | 0.005 | 0.000 | 0.328 | 0.204 | 0.433 |
| SFA | *r* value | -0.055 | 0.449** | 0.422** | 0.660** | -0.011 | -0.045 | -0.035 | -0.045 | -0.011 | 0.883** | 0.494** | 0.332** | -0.044 | 0.422** |
| | *p* value | 0.533 | 0.000 | 0.000 | 0.000 | 0.833 | 0.385 | 0.503 | 0.561 | 0.825 | 0.000 | 0.000 | 0.000 | 0.340 | 0.000 |
| C14:1 | *r* value | 0.115 | 0.416* | 0.014 | 0.240** | 0.311** | 0.040 | 0.296** | 0.058 | -0.054 | 0.131 | 0.033 | -0.016 | 0.003 | 0.014 |
| | *p* value | 0.245 | 0.035 | 0.875 | 0.006 | 0.000 | 0.650 | 0.001 | 0.524 | 0.544 | 0.136 | 0.728 | 0.394 | 0.266 | 0.929 |
| C16:1 | *r* value | -0.062 | 0.053 | 0.119* | 0.376** | 0.371** | 0.078 | 0.359** | -0.291** | -0.074 | 0.277** | 0.100 | 0.075 | -0.051 | 0.119* |
| | *p* value | 0.507 | 0.677 | 0.048 | 0.000 | 0.000 | 0.192 | 0.000 | 0.000 | 0.218 | 0.000 | 0.393 | 0.699 | 0.966 | 0.119 |
| C17:1 | *r* value | -0.038 | -0.147 | -0.261** | -0.229** | -0.031 | -0.176** | -0.103 | 0.078 | 0.074 | -0.230** | -0.236** | -0.255** | 0.082 | -0.261** |
| | *p* value | 0.749 | 0.251 | 0.000 | 0.000 | 0.600 | 0.003 | 0.079 | 0.368 | 0.210 | 0.000 | 0.000 | 0.023 | 0.183 | 0.000 |
| C18:1n9t | *r* value | -0.137 | -0.154 | -0.156 | 0.071 | 0.508** | 0.035 | 0.465** | -0.032 | -0.042 | -0.091 | -0.071 | -0.128 | 0.117 | -0.156 |
| | *p* value | 0.400 | 0.295 | 0.050 | 0.375 | 0.000 | 0.663 | 0.000 | 0.774 | 0.605 | 0.253 | 0.434 | 0.953 | 0.212 | 0.063 |
| C18:1n9c | *r* value | -0.029 | 0.309* | 0.354** | 0.854** | 0.547** | 0.021 | 0.455** | -0.467** | -0.238** | 0.891** | 0.443** | 0.280** | -0.035 | 0.354** |
| | *p* value | 0.746 | 0.013 | 0.000 | 0.000 | 0.000 | 0.688 | 0.000 | 0.000 | 0.000 | 0.000 | 0.000 | 0.000 | 0.447 | 0.000 |
| C20:1n9 | *r* value | 0.617** | 0.515** | 0.377** | 0.333** | 0.097 | 0.346** | 0.242* | -0.130 | -0.101 | 0.278** | 0.421** | 0.115 | -0.150 | 0.377** |
| | *p* value | 0.003 | 0.004 | 0.000 | 0.000 | 0.315 | 0.000 | 0.011 | 0.308 | 0.297 | 0.003 | 0.000 | 0.316 | 0.947 | 0.000 |
| C22:1n9 | *r* value | 0.210* | 0.363 | -0.095 | -0.022 | 0.013 | -0.096 | -0.022 | 0.177 | 0.043 | -0.047 | -0.109 | -0.025 | 0.000 | -0.095 |
| | *p* value | 0.035 | 0.377 | 0.334 | 0.823 | 0.895 | 0.329 | 0.824 | 0.070 | 0.670 | 0.635 | 0.195 | 0.161 | 0.461 | 0.105 |
| MUFA | *r* value | 0.019 | 0.000 | 0.331** | 0.933** | 0.757** | 0.054 | 0.660** | -0.413** | -0.138** | 0.874** | 0.416** | 0.188** | 0.078 | 0.331** |
| | *p* value | 0.833 | 0.999 | 0.000 | 0.000 | 0.000 | 0.295 | 0.000 | 0.000 | 0.007 | 0.000 | 0.000 | 0.000 | 0.254 | 0.000 |
| C18:2n6c | *r* value | 0.099 | 0.249* | 0.775** | 0.605** | 0.074 | 0.535** | 0.274** | -0.182* | -0.108* | 0.638** | 0.955** | 0.243** | 0.159** | 0.775** |

| | | | | | | | | | | | | | | | |
|---|---|---|---|---|---|---|---|---|---|---|---|---|---|---|---|
| | *p* value | 0.260 | 0.047 | 0.000 | 0.000 | 0.165 | 0.000 | 0.000 | 0.017 | 0.042 | 0.000 | 0.000 | 0.000 | 0.000 | 0.000 |
| C18:3n3 | *r* value | 0.104 | 0.183 | 0.461$^{**}$ | 0.345$^{**}$ | 0.037 | 0.360$^{**}$ | 0.180$^{**}$ | -0.138 | -0.097 | 0.365$^{**}$ | 0.191$^{**}$ | 0.899$^{**}$ | -0.624$^{**}$ | 0.461$^{**}$ |
| | *p* value | 0.262 | 0.265 | 0.000 | 0.000 | 0.553 | 0.000 | 0.004 | 0.137 | 0.121 | 0.000 | 0.001 | 0.000 | 0.000 | 0.000 |
| C20:3n6 | *r* value | 0.071 | 0.238 | 0.047 | -0.121 | -0.286$^{**}$ | -0.041 | -0.267$^{**}$ | 0.210 | -0.276$^{**}$ | 0.028 | 0.173 | -0.031 | 0.010 | 0.047 |
| | *p* value | 0.664 | 0.096 | 0.653 | 0.246 | 0.005 | 0.695 | 0.009 | 0.061 | 0.007 | 0.788 | 0.266 | 0.733 | 0.270 | 0.941 |
| C20:4n6 | *r* value | 0.132 | -0.083 | 0.440$^{**}$ | 0.350$^{**}$ | 0.182$^{*}$ | 0.374$^{**}$ | 0.290$^{**}$ | -0.017 | -0.161$^{*}$ | 0.338$^{**}$ | 0.509$^{**}$ | 0.153$^{*}$ | 0.078 | 0.440$^{**}$ |
| | *p* value | 0.143 | 0.742 | 0.000 | 0.000 | 0.017 | 0.000 | 0.000 | 0.856 | 0.037 | 0.000 | 0.000 | 0.541 | 0.050 | 0.001 |
| C20:5n3 | *r* value | 1.000 | -0.030 | 0.333$^{**}$ | 0.126 | 0.039 | 0.344$^{**}$ | 0.158 | -0.067 | -0.164 | 0.078 | 0.174$^{*}$ | 0.366$^{**}$ | -0.236$^{**}$ | 0.333$^{**}$ |
| | *p* value | | 0.955 | 0.000 | 0.148 | 0.656 | 0.000 | 0.070 | 0.502 | 0.063 | 0.377 | 0.203 | 0.262 | 0.489 | 0.090 |
| C22:6n3 | *r* value | -0.030 | 1.000 | 0.391$^{**}$ | 0.125 | -0.295$^{*}$ | 0.156 | -0.237 | 0.088 | -0.332$^{**}$ | 0.322$^{**}$ | 0.100 | 0.462$^{**}$ | -0.513$^{**}$ | 0.391$^{**}$ |
| | *p* value | 0.955 | | 0.001 | 0.322 | 0.017 | 0.215 | 0.057 | 0.572 | 0.007 | 0.009 | 0.098 | 0.265 | 0.603 | 0.001 |
| PUFA | *r* value | 0.333$^{**}$ | 0.391$^{**}$ | 1.000 | 0.641$^{**}$ | 0.088 | 0.874$^{**}$ | 0.457$^{**}$ | -0.273$^{**}$ | 0.065 | 0.563$^{**}$ | 0.857$^{**}$ | 0.609$^{**}$ | -0.176$^{**}$ | 1.000$^{**}$ |
| | *p* value | 0.000 | 0.001 | | 0.000 | 0.089 | 0.000 | 0.000 | 0.000 | 0.210 | 0.000 | 0.000 | 0.000 | 0.015 | 0.000 |
| UFA | *r* value | 0.126 | 0.125 | 0.641$^{**}$ | 1.000 | 0.648$^{**}$ | 0.368$^{**}$ | 0.713$^{**}$ | -0.456$^{**}$ | -0.087 | 0.919$^{**}$ | 0.635$^{**}$ | 0.374$^{**}$ | 0.010 | 0.641$^{**}$ |
| | *p* value | 0.148 | 0.322 | 0.000 | | 0.000 | 0.000 | 0.000 | 0.000 | 0.094 | 0.000 | 0.000 | 0.000 | 0.094 | 0.000 |
| MUFA/SFA | *r* value | 0.039 | -0.295$^{*}$ | 0.088 | 0.648$^{**}$ | 1.000 | 0.116$^{*}$ | 0.897$^{**}$ | -0.409$^{**}$ | -0.141$^{**}$ | 0.392$^{**}$ | 0.132$^{*}$ | -0.019 | 0.131$^{*}$ | 0.088 |
| | *p* value | 0.656 | 0.017 | 0.089 | 0.000 | | 0.024 | 0.000 | 0.000 | 0.006 | 0.000 | 0.173 | 0.553 | 0.375 | 0.267 |
| PUFA/SFA | *r* value | 0.344$^{**}$ | 0.156 | 0.874$^{**}$ | 0.368$^{**}$ | 0.116$^{*}$ | 1.000 | 0.536$^{**}$ | -0.288$^{**}$ | 0.071 | 0.172$^{**}$ | 0.665$^{**}$ | 0.494$^{**}$ | -0.174$^{**}$ | 0.874$^{**}$ |
| | *p* value | 0.000 | 0.215 | 0.000 | 0.000 | 0.024 | | 0.000 | 0.000 | 0.171 | 0.001 | 0.000 | 0.000 | 0.037 | 0.000 |
| UFA/SFA | *r* value | 0.158 | -0.237 | 0.457$^{**}$ | 0.713$^{**}$ | 0.897$^{**}$ | 0.536$^{**}$ | 1.000 | -0.487$^{**}$ | -0.086 | 0.405$^{**}$ | 0.379$^{**}$ | 0.187$^{**}$ | 0.057 | 0.457$^{**}$ |
| | *p* value | 0.070 | 0.057 | 0.000 | 0.000 | 0.000 | 0.000 | | 0.000 | 0.095 | 0.000 | 0.000 | 0.004 | 0.144 | 0.000 |
| SCFA | *r* value | -0.067 | 0.088 | -0.273$^{**}$ | -0.456$^{**}$ | -0.409$^{**}$ | -0.288$^{**}$ | -0.487$^{**}$ | 1.000 | 0.261$^{**}$ | -0.418$^{**}$ | -0.240$^{**}$ | -0.021 | -0.139 | -0.273$^{**}$ |
| | *p* value | 0.502 | 0.572 | 0.000 | 0.000 | 0.000 | 0.000 | 0.000 | | 0.001 | 0.000 | 0.008 | 0.137 | 0.377 | 0.002 |
| MCFA | *r* value | -0.164 | -0.332$^{**}$ | 0.065 | -0.087 | -0.141$^{**}$ | 0.071 | -0.086 | 0.261$^{**}$ | 1.000 | -0.162$^{**}$ | -0.047 | -0.038 | 0.040 | 0.065 |
| | *p* value | 0.063 | 0.007 | 0.210 | 0.094 | 0.006 | 0.171 | 0.095 | 0.001 | | 0.002 | 0.049 | 0.121 | 0.728 | 0.743 |
| LCFA | *r* value | 0.078 | 0.322$^{**}$ | 0.563$^{**}$ | 0.919$^{**}$ | 0.392$^{**}$ | 0.172$^{**}$ | 0.405$^{**}$ | -0.418$^{**}$ | -0.162$^{**}$ | 1.000 | 0.606$^{**}$ | 0.384$^{**}$ | -0.028 | 0.563$^{**}$ |

| | | | | | | | | | | | | | | | |
|---|---|---|---|---|---|---|---|---|---|---|---|---|---|---|---|
| | *p* value | 0.377 | 0.009 | 0.000 | 0.000 | 0.000 | 0.001 | 0.000 | 0.000 | 0.002 | | 0.000 | 0.000 | 0.259 | 0.000 |
| n-6 | *r* value | 0.174* | 0.100 | 0.857** | 0.635** | 0.132* | 0.665** | 0.379** | -0.240** | -0.047 | 0.606** | 1.000 | 0.263** | 0.179** | 0.857** |
| | *p* value | 0.203 | 0.098 | 0.000 | 0.000 | 0.173 | 0.000 | 0.000 | 0.008 | 0.049 | 0.000 | | 0.001 | 0.000 | 0.000 |
| n-3 | *r* value | 0.366** | 0.462** | 0.609** | 0.374** | -0.019 | 0.494** | 0.187** | -0.021 | -0.038 | 0.384** | 0.263** | 1.000 | -0.675** | 0.609** |
| | *p* value | 0.262 | 0.265 | 0.000 | 0.000 | 0.553 | 0.000 | 0.004 | 0.137 | 0.121 | 0.000 | 0.001 | | 0.000 | 0.000 |
| n-6/n-3 | *r* value | -0.236** | -0.513** | -0.176** | 0.010 | 0.131* | -0.174** | 0.057 | -0.139 | 0.040 | -0.028 | 0.179** | -0.675** | 1.000 | -0.176** |
| | *p* value | 0.489 | 0.603 | 0.015 | 0.094 | 0.375 | 0.037 | 0.144 | 0.377 | 0.728 | 0.259 | 0.000 | 0.000 | | 0.593 |
| EFA | *r* value | 0.333** | 0.391** | 1.000** | 0.641** | 0.088 | 0.874** | 0.457** | -0.273** | 0.065 | 0.563** | 0.857** | 0.609** | -0.176** | 1.000 |
| | *p* value | 0.090 | 0.001 | 0.000 | 0.000 | 0.267 | 0.000 | 0.000 | 0.002 | 0.743 | 0.000 | 0.000 | 0.000 | 0.593 | |

Note: *$p < 0.05$, **$p < 0.01$.
