# Supplementary material for: Effects of the Expressions and Variants of the CAST Gene on the Fatty Acid Composition of the Longissimus Thoracis Muscle of Grazing Sonid Sheep
Source: Animals (Basel). 2023 Jan 4;13(2):195. doi: 10.3390/ani13020195 (PMC9855194; doi:10.3390/ani13020195)
Supplement: Supplementary file 1 [file animals-13-00195-s001.zip › animals-2068675-supplementary/Table S3. Statistical difference of the CAST gene expression between different tissues in Sonid sheep.pdf]

Table S3. Statistical difference of the *CAST* gene expression between different tissues in Sonid sheep.

	**Lung**	**Subcutaneous fat**	**Kidney**	**Large intestine**	**Small intestine**	**Semitendinosus muscle**	**Longissimus muscle**	**Stomach**
Heart	***	***	***	***	***	***	***	***
Lung		***	***	**	***	***	***	n.s.
Subcutaneous fat			***	***	***	***	***	***
Kidney				***	***	***	**	***
Large intestine					***	***	***	*
Small intestine						***	***	***
Semitendinosus muscle							***	***
Longissimus muscle								***

Note: n.s.: non-significant, *$p < 0.05$, **$p < 0.01$, **$p < 0.001$.
