# Supplementary material for: Effects of the Expressions and Variants of the CAST Gene on the Fatty Acid Composition of the Longissimus Thoracis Muscle of Grazing Sonid Sheep
Source: Animals (Basel). 2023 Jan 4;13(2):195. doi: 10.3390/ani13020195 (PMC9855194; doi:10.3390/ani13020195)
Supplement: Supplementary file 1 [file animals-13-00195-s001.zip › animals-2068675-supplementary/Table S4. Genotypic frequencies, allelic frequencies and diversity parameters of four mutations in CAST of Sonid population.pdf]

Table S4. Genotypic frequencies, allelic frequencies, and diversity parameters of four mutations in *CAST* of Sonid population.

SNPs	Genotype frequency			Allele frequency		Diversity parameter				
						H_o	H_e	n_e	PIC	χ^2 (HWE[2])
c.646G>C (G216R)	GG	GC	CC	G	C					
	0.770	0.214	0.016	0.877	0.123	0.784	0.216	1.275	0.192	0.018
c.1210C>T (R404C)	CC	CT	TT	C	T					
	0.669	0.302	0.029	0.820	0.180	0.705	0.295	1.419	0.252	0.185
c.1437G>A (479T)	GG	GA	AA	G	A					
	0.762	0.222	0.016	0.873	0.127	0.778	0.222	1.285	0.197	0.002
c.2097C>T (699G)	CC	CT	TT	C	T					
	0.759	0.235	0.005	0.877	0.123	0.784	0.216	1.275	0.192	3.146

Note: H_o: observed heterozygosity, H_e: expected heterozygosity, n_e: effective allele numbers, PIC: polymorphism information content, HWE: Hardy-Weinberg equilibrium. The classification was conducted according to the PIC values (PIC value < 0.25, low polymorphism; 0.25 < PIC value < 0.5, moderate polymorphism; and PIC value > 0.5, high polymorphism). No Hardy-Weinberg departure was detected from the obtained genotype frequencies.
