# Supplementary material for: Effects of the Expressions and Variants of the CAST Gene on the Fatty Acid Composition of the Longissimus Thoracis Muscle of Grazing Sonid Sheep
Source: Animals (Basel). 2023 Jan 4;13(2):195. doi: 10.3390/ani13020195 (PMC9855194; doi:10.3390/ani13020195)
Supplement: Supplementary file 1 [file animals-13-00195-s001.zip › animals-2068675-supplementary/Table S5. Linkage disequilibrium as measured by D' and r2 among four mutations in the CAST.pdf]

Table S5. Linkage disequilibrium as measured by D' and r^2 among four mutations in the *CAST*.

SNPs	c.646G>C	c.1210C>T	c.1437G>A
c.1210C>T	D' = 1.000/r^2 = 0.639		
c.1437G>A	D' = 1.000/r^2 = 0.964	D' = 1.000/r^2 = 0.663	
c.2097C>T	D' = 0.799/r^2 = 0.639	D' = 0.771/r^2 = 0.380	D' = 0.797/r^2 = 0.613
