# Supplementary material for: Effects of the Expressions and Variants of the CAST Gene on the Fatty Acid Composition of the Longissimus Thoracis Muscle of Grazing Sonid Sheep
Source: Animals (Basel). 2023 Jan 4;13(2):195. doi: 10.3390/ani13020195 (PMC9855194; doi:10.3390/ani13020195)
Supplement: Supplementary file 1 [file animals-13-00195-s001.zip › animals-2068675-supplementary/Table S6. Associations of CAST variants with fatty acid composition in longissimus thoracis muscles in Sonid sheep.pdf]

Table S6. Associations of *CAST* variants with fatty acid composition in longissimus thoracis muscles in Sonid sheep.

Fatty acid composition	c.1210C>T (R404C)			c.1437G>A in LD-M		c.2097C>T	
	Genotype			Genotype		Genotype	
	CC (253)	CT (114)	TT (11)	GG (288)	GA (84)	CC (287)	CT (89)
C4:0	1.84 ± 0.08	1.90 ± 0.12	1.83 ± 0.22	1.86 ± 0.07	1.83 ± 0.14	1.86 ± 0.07	1.77 ± 0.16
C6:0	0.85 ± 0.07	0.66 ± 0.08	0.64 ± 0.14	0.83 ± 0.07	0.74 ± 0.09	0.80 ± 0.06	0.67 ± 0.11
C11:0	0.46 ± 0.01	0.45 ± 0.02	0.51 ± 0.06	0.46 ± 0.01	0.45 ± 0.02	0.45 ± 0.01	0.47 ± 0.02
C13:0	0.64 ± 0.05	0.62 ± 0.07	0.64 ± 0.19	0.64 ± 0.04	0.63 ± 0.11	0.64 ± 0.04	0.61 ± 0.08
C15:0	0.75 ± 0.03	0.80 ± 0.04	0.94 ± 0.13	0.75 ± 0.02	0.82 ± 0.05	0.75 ± 0.02	0.83 ± 0.05
C16:0	17.05 ± 0.23	16.30 ± 0.28	18.10 ± 0.98	17.01 ± 0.21	16.30 ± 0.34	16.92 ± 0.20	16.67 ± 0.38
C17:0	0.69 ± 0.12	0.72 ± 0.07	0.74 ± 0.16	0.71 ± 0.10	0.69 ± 0.03	0.71 ± 0.09	0.67 ± 0.04
C21:0	0.54 ± 0.03	0.67 ± 0.22	0.52 ± 0.03	0.63 ± 0.08	0.59 ± 0.05	0.62 ± 0.08	0.50 ± 0.04
C22:0	0.65 ± 0.03	0.67 ± 0.06	0.72 ± 0.00	0.66 ± 0.03	0.58 ± 0.00	0.66 ± 0.03	0.54 ± 0.10
C23:0	0.57 ± 0.02	0.54 ± 0.02	0.57 ± 0.23	0.56 ± 0.01	0.56 ± 0.03	0.56 ± 0.01	0.55 ± 0.03
C24:0	0.53 ± 0.02	0.53 ± 0.02	0.49 ± 0.22	0.53 ± 0.02	0.54 ± 0.03	0.53 ± 0.02	0.54 ± 0.03
C14:1	0.77 ± 0.12	0.63 ± 0.06	0.50 ± 0.06	0.76 ± 0.11	0.67 ± 0.07	0.76 ± 0.10	0.52 ± 0.07
C16:1	0.97 ± 0.10	0.81 ± 0.03	0.91 ± 0.09	0.96 ± 0.08	0.79 ± 0.03	0.94 ± 0.08	0.85 ± 0.03
C17:1	0.73 ± 0.03	0.81 ± 0.03	0.70 ± 0.11	0.74 ± 0.03	0.83 ± 0.03	0.74 ± 0.03	0.81 ± 0.03
C20:1n9	0.70 ± 0.02	0.71 ± 0.02	0.69 ± 0.15	0.70 ± 0.02	0.71 ± 0.03	0.72 ± 0.02	0.65 ± 0.02
C22:1n9	0.55 ± 0.03	0.63 ± 0.04	0.54 ± 0.08	0.56 ± 0.03	0.63 ± 0.05	0.56 ± 0.03	0.62 ± 0.04
MUFA	21.62 ± 0.32	20.72 ± 0.41	21.23 ± 1.04	21.57 ± 0.30	20.70 ± 0.43	21.47 ± 0.30	21.02 ± 0.47
C18:2n6c	4.44 ± 0.07	4.53 ± 0.12	5.30 ± 0.29	4.43 ± 0.07	4.64 ± 0.15	4.44 ± 0.07	4.65 ± 0.15
C20:3n6	0.45 ± 0.02	0.45 ± 0.03	0.50 ± 0.05	0.45 ± 0.02	0.46 ± 0.03	0.45 ± 0.02	0.47 ± 0.04
C20:4n6	0.56 ± 0.04	0.49 ± 0.05	0.64 ± 0.08	0.55 ± 0.03	0.51 ± 0.05	0.54 ± 0.03	0.57 ± 0.06
C20:5n3	0.56 ± 0.03	0.55 ± 0.04	0.48 ± 0.10	0.55 ± 0.02	0.57 ± 0.05	0.57 ± 0.02	0.51 ± 0.04

C22:6n3	0.44 ± 0.02	0.41 ± 0.02	0.37 ± 0.00	0.44 ± 0.02	0.40 ± 0.03	0.44 ± 0.02	0.38 ± 0.03
PUFA	8.21 ± 0.14	7.98 ± 0.20	8.80 ± 0.64	8.16 ± 0.13	8.11 ± 0.26	8.19 ± 0.13	8.11 ± 0.26
UFA	29.83 ± 0.39	28.70 ± 0.51	30.03 ± 1.52	29.73 ± 0.37	28.81 ± 0.57	29.66 ± 0.36	29.13 ± 0.62
MUFA/SFA	0.60 ± 0.01	0.60 ± 0.01	0.58 ± 0.03	0.60 ± 0.01	0.60 ± 0.01	0.60 ± 0.01	0.60 ± 0.01
PUFA/SFA	0.23 ± 0.00	0.23 ± 0.01	0.24 ± 0.02	0.23 ± 0.00	0.24 ± 0.01	0.23 ± 0.00	0.23 ± 0.01
UFA/SFA	0.83 ± 0.01	0.82 ± 0.01	0.82 ± 0.05	0.82 ± 0.01	0.84 ± 0.02	0.83 ± 0.01	0.83 ± 0.02
SCFA	1.84 ± 0.08	1.90 ± 0.12	1.83 ± 0.22	1.86 ± 0.07	1.83 ± 0.14	1.86 ± 0.07	1.77 ± 0.16
MCFA	2.06 ± 0.04	2.04 ± 0.05	2.02 ± 0.15	2.07 ± 0.03	2.11 ± 0.06	2.03 ± 0.03	2.08 ± 0.06
LCFA	62.05 ± 0.68	59.55 ± 0.86	63.02 ± 2.44	62.02 ± 0.63	59.32 ± 0.97	61.68 ± 0.61	60.32 ± 1.12
EFA	8.21 ± 0.14	7.98 ± 0.20	8.80 ± 0.64	8.16 ± 0.13	8.11 ± 0.26	8.19 ± 0.13	8.11 ± 0.26

Note: Values are shown as the means ± standard error.
