# Supplementary material for: Effects of the Expressions and Variants of the CAST Gene on the Fatty Acid Composition of the Longissimus Thoracis Muscle of Grazing Sonid Sheep
Source: Animals (Basel). 2023 Jan 4;13(2):195. doi: 10.3390/ani13020195 (PMC9855194; doi:10.3390/ani13020195)
Supplement: Supplementary file 1 [file animals-13-00195-s001.zip › animals-2068675-supplementary/Table S7. Associations between haplotypes of CAST and fatty acid composition in longissimus thoracis muscles in Sonid sheep.pdf]

Table S7. Associations between haplotypes of *CAST* and fatty acid composition in longissimus thoracis muscles in Sonid sheep.

Fatty acid composition	Genotype of combination (Number)		
	**H1H1 (238) (GGCCGGCC)**	**H1H2 (64) (GCCTGACT)**	**H1H3 (35) (GGCTGGCC)**
C4:0	1.85 ± 0.08	1.91 ± 0.19	2.01 ± 0.24
C6:0	0.85 ± 0.07	0.72 ± 0.14	0.68 ± 0.16
C10:0	0.31 ± 0.01	0.38 ± 0.03	0.30 ± 0.04
C11:0	0.46 ± 0.01	0.47 ± 0.02	0.44 ± 0.04
C13:0	0.64 ± 0.05	0.60 ± 0.10	0.62 ± 0.06
C14:0	1.55 ± 0.04	1.39 ± 0.07	1.53 ± 0.09
C15:0	0.76 ± 0.03	0.85 ± 0.06	0.77 ± 0.07
C16:0	17.04 ± 0.23	16.29 ± 0.40	16.72 ± 0.47
C17:0	0.69 ± 0.12	0.66 ± 0.04	0.79 ± 0.21
C18:0	9.21 ± 0.13	8.58 ± 0.24	9.23 ± 0.33
C22:0	0.65 ± 0.03	0.38 ± 0.00	0.72 ± 0.05
C23:0	0.56 ± 0.02	0.53 ± 0.03	0.51 ± 0.03
C24:0	0.53 ± 0.02	0.54 ± 0.03	0.52 ± 0.04
SFA	36.08 ± 0.34	34.41 ± 0.60	36.73 ± 0.78
C14:1	0.78 ± 0.13	0.51 ± 0.10	0.66 ± 0.11
C16:1	0.97 ± 0.10	0.8 ± 0.04	0.87 ± 0.05
C17:1	0.73 ± 0.04	0.85 ± 0.04	0.77 ± 0.06
C18:1n9t	1.72 ± 0.14	2.45 ± 0.41	1.69 ± 0.23
C18:1n9c	16.16 ± 0.28	14.76 ± 0.52	15.76 ± 0.85
C20:1n9	0.69 ± 0.03	0.63 ± 0.03	0.71 ± 0.04
C22:1n9	0.55 ± 0.03	0.65 ± 0.05	0.60 ± 0.09
MUFA	21.60 ± 0.33	20.65 ± 0.53	21.06 ± 0.87
C18:2n6c	4.45 ± 0.08	4.62 ± 0.19	4.40 ± 0.15
C20:3n6	0.45 ± 0.02	0.46 ± 0.05	0.42 ± 0.07
C20:4n6	0.55 ± 0.04	0.48 ± 0.06	0.51 ± 0.08
C20:5n3	0.57 ± 0.03	0.54 ± 0.05	0.48 ± 0.06
C22:6n3	0.44 ± 0.02	0.38 ± 0.04	0.44 ± 0.04
PUFA	8.23 ± 0.15	8.00 ± 0.31	7.84 ± 0.27
UFA	29.83 ± 0.4	28.65 ± 0.69	28.90 ± 1.01
MUFA/SFA	0.60 ± 0.01	0.60 ± 0.02	0.57 ± 0.02
PUFA/SFA	0.23 ± 0.00	0.23 ± 0.01	0.21 ± 0.01
UFA/SFA	0.83 ± 0.01	0.83 ± 0.02	0.79 ± 0.02
SCFA	1.85 ± 0.08	1.91 ± 0.19	2.01 ± 0.24
MCFA	2.06 ± 0.04	2.16 ± 0.07	2.11 ± 0.12
LCFA	62.00 ± 0.68	58.99 ± 1.18	61.51 ± 1.66
n-6	5.45 ± 0.09	5.56 ± 0.22	5.33 ± 0.21
EFA	8.23 ± 0.15	8.00 ± 0.31	7.84 ± 0.27

Note: Values are shown as the means ± standard error.
